# AUTS2 Regulation of Synapses for Proper Synaptic Inputs and Social Communication

**DOI:** 10.1016/j.isci.2020.101183

**Published:** 2020-05-18

**Authors:** Kei Hori, Kunihiko Yamashiro, Taku Nagai, Wei Shan, Saki F. Egusa, Kazumi Shimaoka, Hiroshi Kuniishi, Masayuki Sekiguchi, Yasuhiro Go, Shoji Tatsumoto, Mitsuyo Yamada, Reika Shiraishi, Kouta Kanno, Satoshi Miyashita, Asami Sakamoto, Manabu Abe, Kenji Sakimura, Masaki Sone, Kazuhiro Sohya, Hiroshi Kunugi, Keiji Wada, Mitsuhiko Yamada, Kiyofumi Yamada, Mikio Hoshino

**Affiliations:** 1 Department of Biochemistry and Cellular Biology, National Institute of Neuroscience, NCNP, Tokyo 187-8502, Japan; 2 Department of Neuropsychopharmacology and Hospital Pharmacy, Nagoya University Graduate School of Medicine, Nagoya 466-8560, Japan; 3 Department of Neuropsychopharmacology, National Institute of Mental Health, NCNP, Tokyo, 187-8502, Japan; 4 Department of Degenerative Neurological Diseases, National Institute of Neuroscience, NCNP, Tokyo, 187-8502, Japan; 5 Cognitive Genomics Research Group, Exploratory Research Center on Life and Living Systems, National Institutes of Natural Sciences, Okazaki, Aichi 444-8585, Japan; 6 School of Life Science, SOKENDAI (The Graduate University for Advanced Studies), Okazaki, Aichi 444-8585, Japan; 7 Department of Physiological Sciences, National Institute for Physiological Sciences, Okazaki, Aichi 444-8585, Japan; 8 Department of Biomolecular Science, Faculty of Science, Toho University, Chiba 274-8510, Japan; 9 Department of Humanities, Faculty of Law, Economics and the Humanities, Kagoshima University, Kagoshima 890-0065, Japan; 10 Department of Cellular Neurobiology, Brain Research Institute, Niigata University, Nigata 951-8585, Japan; 11 Department of Mental Disorder Research, National Institute of Neuroscience, NCNP, Tokyo 187-8502, Japan

**Keywords:** Neuroscience, Behavioral Neuroscience, Molecular Neuroscience, Transcriptomics

## Abstract

Impairments in synapse development are thought to cause numerous psychiatric disorders. *Autism susceptibility candidate 2* (*AUTS2*) gene has been associated with various psychiatric disorders, such as autism and intellectual disabilities. Although roles for AUTS2 in neuronal migration and neuritogenesis have been reported, its involvement in synapse regulation remains unclear. In this study, we found that excitatory synapses were specifically increased in the *Auts2*-deficient primary cultured neurons as well as *Auts2* mutant forebrains. Electrophysiological recordings and immunostaining showed increases in excitatory synaptic inputs as well as c-fos expression in *Auts2* mutant brains, suggesting that an altered balance of excitatory and inhibitory inputs enhances brain excitability. *Auts2* mutant mice exhibited autistic-like behaviors including impairments in social interaction and altered vocal communication. Together, these findings suggest that AUTS2 regulates excitatory synapse number to coordinate E/I balance in the brain, whose impairment may underlie the pathology of psychiatric disorders in individuals with *AUTS2* mutations.

## Highlights

• AUTS2 regulates excitatory synapse number in forebrain pyramidal neurons
• Loss of *Auts2* leads to increased spine formation in development and adulthood
• Loss of *Auts2* alters the balance of excitatory and inhibitory synaptic inputs
• *Auts2* mutant mice exhibit cognitive and sociobehavioral deficits

## Graphical Abstract

**Figure:**
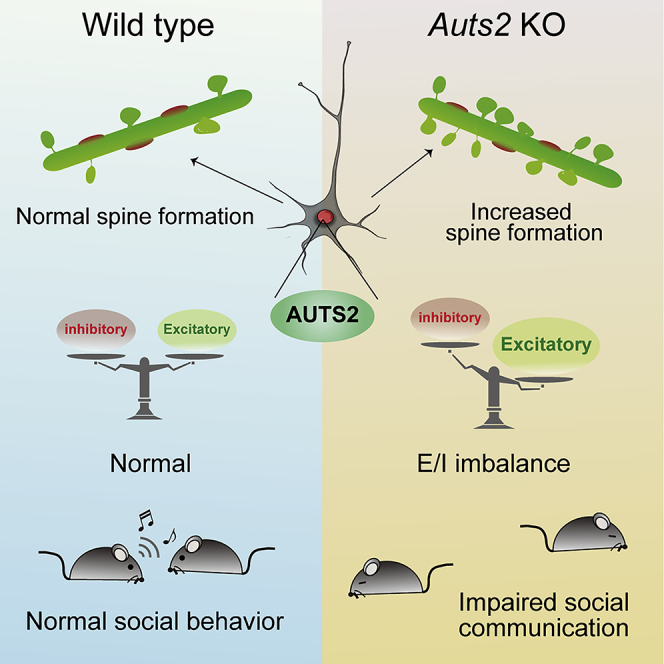

## Introduction

Synapses form the basis for the neuronal network and brain function. Development of synapses, synaptogenesis, is precisely regulated by genetic programs as well as synaptic activities. Even after establishment of the fundamental brain structures, synapses are dynamically formed and eliminated in response to neuro-environmental stimuli (Holtmaat and Svoboda, 2009). However, maintenance of the number of synapses within a certain range, comprising the synapse homeostasis, assures neuronal homeostasis (Davis, 2013, Tien and Kerschensteiner, 2018, Wefelmeyer et al., 2016). It has been proposed that failure of either synapse or neuronal homeostasis results in various neuropsychiatric disorders (Bourgeron, 2009, Ramocki and Zoghbi, 2008). Consistent with this, postmortem pathological studies have revealed that aberrant regulation of dendritic spine number as well as structural abnormalities of spines were observed in patients with numerous psychiatric disorders such as autism spectrum disorders (ASDs), schizophrenia, and neurodegenerative diseases (Hutsler and Zhang, 2010, Penzes et al., 2011, Tang et al., 2014). Thus, appropriate regulation of synaptogenesis as well as synapse homeostasis is critical for normal healthy brain function; however, its molecular machinery remains elusive.

*Autism susceptibility candidate 2* (*AUTS2*) (also termed “activator of transcription and developmental regulator”) located on human chromosome 7q11.22 has been initially identified as a possible ASD risk gene in a study that reported a *de novo* balanced translocation in monozygotic twin patients with ASDs (Sultana et al., 2002). Thereafter, structural variants that disrupt the *AUTS2* locus have been identified in the patients with not only autism but also other neuropathological conditions including intellectual disabilities (IDs), schizophrenia, attention deficit hyperactivity disorder (ADHD), dyslexia, and epilepsy, as well as brain malformation and craniofacial abnormalities (Amarillo et al., 2014, Bakkaloglu et al., 2008, Ben-David et al., 2011, Beunders et al., 2013, Elia et al., 2010, Hori and Hoshino, 2017, Jolley et al., 2013, Kalscheuer et al., 2007, Oksenberg and Ahituv, 2013, Talkowski et al., 2012, Zhang et al., 2014). In addition, *AUTS2* has been recently implicated as a potential gene in human-specific evolution (Oksenberg and Ahituv, 2013, Oksenberg et al., 2013).

We previously reported that the cytoplasmic AUTS2 acts as an upstream regulator for Rho family small GTPases, Rac1 and Cdc42, in reorganizing actin cytoskeleton (Hori et al., 2014). AUTS2 activates Rac1 to induce lamellipodia while downregulating CDC42 to suppress filopodia. In addition to these functions, Gao et al. showed that nuclear AUTS2 binds to and neutralizes the transcriptional repressor activity of Polycomb group (PcG) protein complex 1 (PRC1) and activates some gene transcription by recruiting the histone acetyltransferase P300 into the complex (Gao et al., 2014).

In the developing mouse brain, *Auts2* expression starts from early embryonic stages in multiple regions of the central nervous system, but particularly strong prenatal expression is observed in the regions associated with higher brain functions including neocortex, hippocampus, and cerebellum (Bedogni et al., 2010). We previously demonstrated that the AUTS2-Rac1 signaling pathway is required for neuronal migration and subsequent neurite formation in the developing cerebral cortex (Hori et al., 2014). However, even at postnatal and adult stages, AUTS2 expression is maintained in various types of neurons (Bedogni et al., 2010). Although this late-stage expression raised the possibility that AUTS2 may also be involved in later neurodevelopmental processes, such as synaptogenesis and synaptic homeostasis, its involvement in synapse regulation remains unknown.

In human patients, *AUTS2* mutations are associated with a variety of psychiatric diseases, such as ASD, schizophrenia, depression, intellectual disabilities, and language disability. Although the underlying pathways to evoke this wide range of disorders have not been clarified, one possible mechanism is that different types of gene disruption may cause distinct types of disorders. *AUTS2* is a very large gene with multiple exons and many types of gene mutations, such as deletion, duplication, single nucleotide change, and chromosomal translocation, have been reported in humans (Hori and Hoshino, 2017, Oksenberg and Ahituv, 2013).

In this study, we show that AUTS2 coordinates excitation/inhibition balance by restricting the number of excitatory synapses during development as well as at post-developmental stages. Targeted disruption of *Auts2* resulted in excessive numbers of excitatory synapses without affecting inhibitory ones. Consistent with this, electrophysiological analyses showed that excitatory but not inhibitory inputs increased in the mutant hippocampal neurons where strong c-Fos signals were detected, suggesting impairment in the excitatory and inhibitory coordination in that region. Behavioral analyses on *Auts2* heterozygous mutant mice revealed abnormalities in social interaction and altered vocal communication as well as the defects in recognition. Thus, our data suggest that AUTS2 regulates synapse homeostasis by restricting the number of excitatory synapses without affecting inhibitory ones and that loss of AUTS2 function leads to impaired excitatory and inhibitory coordination that may underlie the pathogenesis of some psychiatric illnesses.

## Results

### *Auts2* Restricts the Number of Excitatory Synapses *In Vitro*

**Figure 1:**
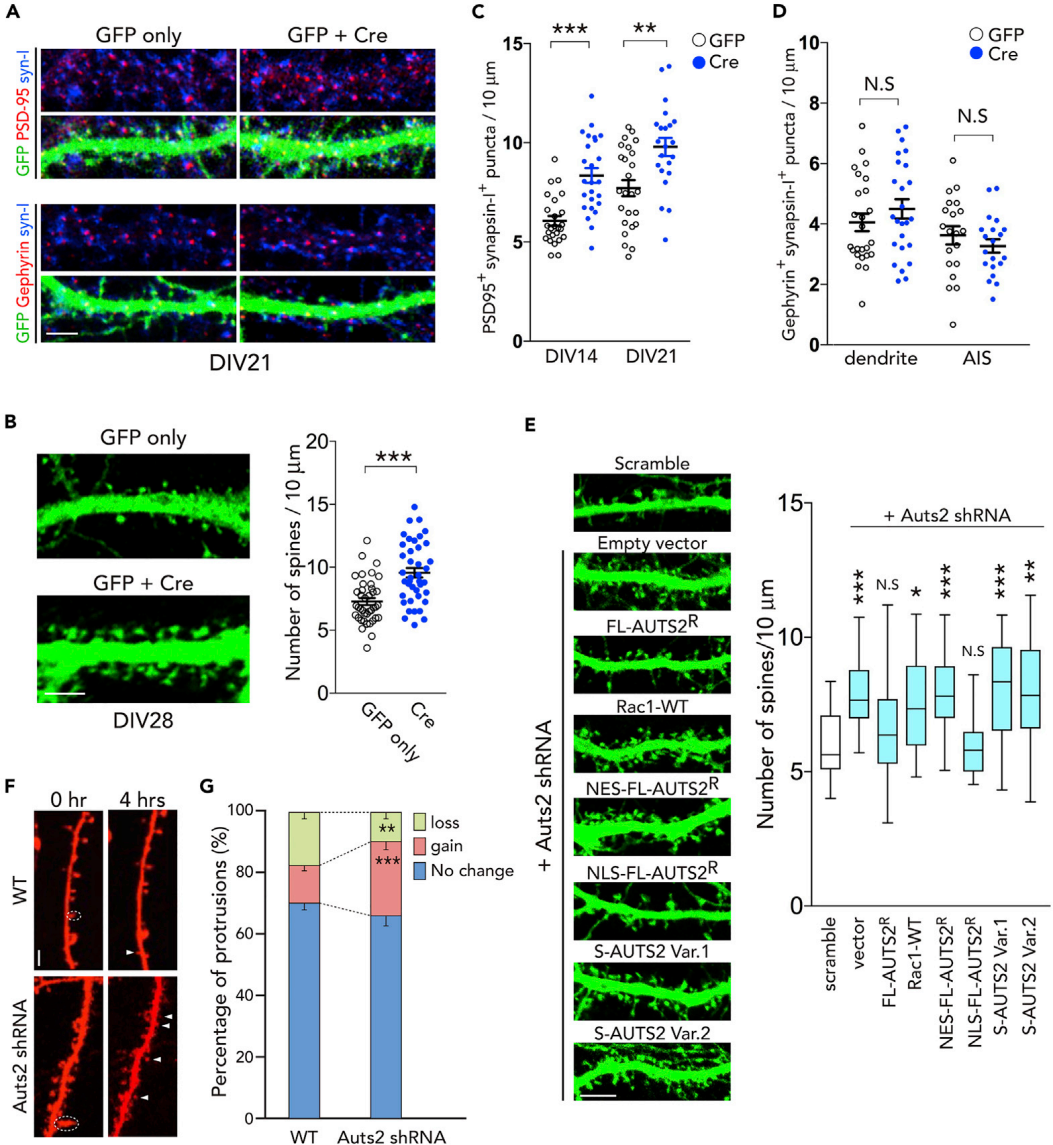
Loss of *Auts2* Induces Excessive Excitatory Synapse Formation (A) Primary hippocampal neurons derived from *Auts2*^*flox/flox*^ homozygotes at DIV21 were immunolabeled with anti-GFP (green), anti-synapsin I (blue) and anti-PSD-95 or Gephyrin (magenta). Neurons were co-electroporated with control or Cre plus GFP expression vector at DIV0. (B) Dendritic spines were increased in *Auts2* KO neurons (GFP + Cre) at DIV28. The graph shows the density of dendritic spines in the GFP-positive neurons (n = 40 dendrites from 20 neurons). (C) The number of PSD-95 puncta colocalized with or adjacent to synapsin-I puncta in GFP-positive cells was measured at DIV14 and 21 (DIV14, n = 28 dendrites; DIV21, n = 51 dendrites of 15–22 neurons). (D) The number of Gephyrin-positive puncta colocalized with Gephyrin on the dendrites and axon initiation sites (AIS) were measured at DIV21 (n = 25 dendrites and n = 20 AIS of 20 neurons). (E) WT primary hippocampal neurons were co-electroporated with *Auts2*-shRNA and the indicated expression vectors and analyzed at DIV22–24. To visualize the neurons, GFP vector was co-electroporated. Graph shows the density of dendritic spines (n = 19–20 dendrites). Expression of the shRNA-resistant FL-AUTS2 (FL-AUTS2^R^) or nuclear-localized form AUTS2 (NLS-AUTS2^R^) in *Auts2*-knockdown neurons rescues the aberrant spine formation. (F) WT mouse hippocampal neurons at DIV16 expressed with mRFP only (WT) or mRFP plus Auts2 shRNA vector were imaged at the beginning (0 h) and 4 h after the analysis (dashed white circle, spine eliminated; white arrowheads, spines formed). (G) Gain and loss of dendritic protrusions (including spines and filopodia) in WT and *Auts2* knockdown neurons were analyzed during a 6-h time window at DIV16–17 (WT, n = 7 neurons; *Auts2* shRNA, n = 10 neurons). Data are presented as mean ± SEM and box-and-whisker plots (medians with interquartile range, minimum, and maximum values are represented). Quantifications in (B)–(E) and (G) represent data from three independent experiments. ∗p < 0.05, ∗∗p < 0.01, ∗∗∗p < 0.001, N.S, not significant. (B–D) Unpaired t test; (E) one-way ANOVA with Dunnett's post hoc test; (G) Mann-Whitney U test. Scale bars represent 10 μm.

To investigate the involvement of AUTS2 in synapse formation, we utilized primary cultured hippocampal neurons from homozygous *Auts2-floxed* (*Auts2*^*flox/flox*^) embryos. Most excitatory synapses in mammalian brain are formed on dendritic spines (Bhatt et al., 2009). We confirmed that, at 21 days *in vitro* (DIV21), most PSD-95 (excitatory postsynapse marker) signals were observed on the spine heads (Figure 1A).

Deletion of *Auts2* was carried out by co-introducing GFP with the Cre recombinase expression vector into the *Auts2*^*flox/flox*^ primary hippocampal neurons. Consistent with our previous report, loss of *Auts2* resulted in the impairment of dendrite development, as shown by decreased total dendritic length (∗∗p = 0.003, Figures S1A and S1B). Furthermore, Sholl analysis revealed that the *Auts2*-deficient neurons exhibited a lower dendritic arbor complexity compared with the control neurons (∗∗p = 0.008, Figure S1C) (Hori et al., 2014).

Immunostaining revealed that the *Auts2*-deficient neurons (*Auts2*^*del8/del8*^ neurons) exhibited a significant increase in the density of dendritic spines compared with the control neurons at DIV28 (∗∗∗p < 0.001, Figure 1B). Consistent with the increased dendritic spines, *Auts2*-deficient neurons harbored a larger number of excitatory synapses defined as puncta double-positive for PSD-95 and presynaptic marker synapsin-I than the control at DIV21 (∗∗p = 0.001, Figures 1A and 1C). The larger number of excitatory synapses were already evident at an early culture stage (DIV14) in the mutant neurons (∗∗∗p < 0.001, Figure 1C). Interestingly, the number of inhibitory postsynapse marker, Gephyrin-positive puncta on the dendrites (p = 0.085) and axon initial segment (AIS) (p = 0.343) as well as the cell somas (WT, 0.086 ± 0.026/μm^2^ (n = 20); KO, 0.090 ± 0.022/μm^2^ (n = 20); data mean ± SEM, p = 0.579, unpaired t test) was not different between the control and *Auts2*-deficient neurons (Figures 1A and 1D). These findings suggest that *Auts2* in postsynaptic cells restricts excessive excitatory synapse formation without affecting inhibitory synapses.

We further observed the development of dendritic spines at different stages in culture (Figure S2A). In control neurons, filopodia were predominantly formed during the first week of culture but gradually decreased from 2 to 4 weeks, with increasing spine formation during the same period. During the first week of culture, *Auts2* mutant neurons had a similar number of protrusions including filopodia and spines as control neurons (Figure S2A: p = 0.300 for filopodia, p = 0.321 for spine). At later stages, however, larger numbers of dendritic spines as well as filopodia were continuously formed in the *Auts2* mutant neurons compared with the control neurons (Figure S2A: DIV14, ∗∗∗p < 0.001 for filopodia, ∗∗∗p < 0.001 for spine; DIV21, ∗∗∗p < 0.001 for filopodia, ∗∗∗p < 0.001 for spine; DIV28, ∗p = 0.039 for filopodia, ∗∗∗p < 0.001 for spine). The *Auts2*-deficient neurons, however, exhibited the same extent of spine maturation with that of WT neurons, as depicted by the spine maturity index (Figure S2B: DIV7, p = 0.220; DIV14, p = 0.664; DIV21, p = 0.903; DIV28, p = 0.595) as well as the spine size (Figure S2C: p = 0.5903 for spine length, p = 0.358 for spine). Furthermore, we observed no significant difference in the PSD-95 puncta size between the control and *Auts2* mutant neurons (p = 0.794, Figure S2D). These results suggest that loss of *Auts2* does not influence the maturation of dendritic spines.

Next, we introduced the expression vectors for AUTS2 isoforms or possible AUTS2 downstream factors into the *Auts2*-knockdown neurons (Hori et al., 2014). We first confirmed that knockdown of *Auts2* well recapitulated aberrant spine formation as observed in *Auts2* KO neurons (Figure 1E: one-way ANOVA, p < 0.001, F_(6,133)_ = 1.781; Dunnett's post hoc test, ∗∗∗p < 0.001). This abnormality was restored by co-expression of the shRNA-resistant full-length AUTS2 (FL-AUTS2^R^), indicating that excess spine formation is the result of specific knockdown of *Auts2* (p = 0.795, Figure 1E). We have previously demonstrated that a cytoplasmic AUTS2-Rac1 signaling pathway is required for neuronal migration in the developing cerebral cortex (Hori et al., 2014). In that study, defective cortical neuronal migration in *Auts2* KO mice was shown to be rescued by introduction of either NES (nuclear export sequence)-tagged FL-AUTS2^R^ (NES-FL-AUTS2^R^) (Figure S2E) or wild-type Rac1 (Rac1-WT). Overexpression of these proteins, however, failed to rescue the aberrant spine formation evoked by *Auts2* knockdown (Figure 1E: ∗p = 0.013 for Rac1-WT and ∗∗p < 0.001 for NES-FL-AUTS2^R^). In contrast, introduction of NLS (nuclear localization signal)-tagged FL-AUTS2^R^ (NLS-FL-AUTS2^R^) (Figure S2E) was able to rescue the spine number to levels comparable with that of control neurons (p = 0.999, Figure 1E), whereas the C-terminal AUTS2 short isoforms (S-AUTS2-var.1 and 2) (Figure S3B), which are exclusively localized in nuclei (Hori et al., 2014), were not able to rescue the phenotype (Figure 1E: ∗∗∗p = 0.001 for S-AUTS2-var.1, ∗∗p = 0.008 for S-AUTS2-var.2). These results indicate that nuclear FL-AUTS2 is involved in the control of spine number.

In *Auts2*^*del8/del8*^ brains, expression of FL-AUTS2 and S-AUTS2-var.1 proteins is eliminated, whereas another C-terminal AUTS2 short isoform variant 2 (S-AUTS2-var.2) is increased (Hori et al., 2014), raising a possibility that aberrant synapses in the primary *Auts2*^*del8/del8*^ hippocampal culture are caused by the overexpression of S-AUTS2-var.2. However, overexpression of S-AUTS2-var.2 into wild-type primary hippocampal neurons did not affect the number and morphology of spines (Figure S2F: one-way ANOVA, p = 0.521), suggesting that the formation of aberrant number of spines in *Auts2* mutant neurons was not due to a gain-of-function effect by increased AUTS2 short isoform expression. Similarly, we also found that FL-AUTS2 or S-AUTS2-var.1 did not affect the spine number (Figure S2F).

Next, we performed live imaging to observe the dynamics of dendritic protrusions including spines and filopodia at DIV16–17. During a 6-h time window, neurons expressing the *Auts2* shRNA exhibited a higher rate of protrusion gain (∗∗∗p < 0.001) and a lower rate of protrusion loss (∗∗p = 0.002) compared with WT neurons (Figures 1F and 1G). Compared with the fixed neurons, a higher number of protrusions were formed in the *Auts2*-knockdown living neurons during the time-lapse recording (Figure 1G). This may be attributed to the difference in experimental conditions. Alternatively, the exposure to laser might have caused damage to living neurons during the time-lapse recording, which may affect the dynamics of cell protrusions.

Altogether, these *in vitro* experiments suggest that AUTS2 restricts the number of excitatory synapses, while not affecting inhibitory neurons.

### Loss of *Auts2* Results in Excessive Dendritic Spines *In Vivo*

To assess the involvement of AUTS2 in the regulation of dendritic spine formation *in vivo*, we generated forebrain-specific *Auts2* conditional KO mice by crossing *Auts2-floxed* mice with *Emx1*^*Cre*^ mice (Iwasato et al., 2000) (Figure S3A and Table S1) and examined brain tissues by Golgi staining to visualize dendrite morphology. Immunoblotting confirmed that expression of FL-AUTS2 protein in the mutant cerebral cortex was successfully eliminated (arrow in Figure S3C).

**Figure 2:**
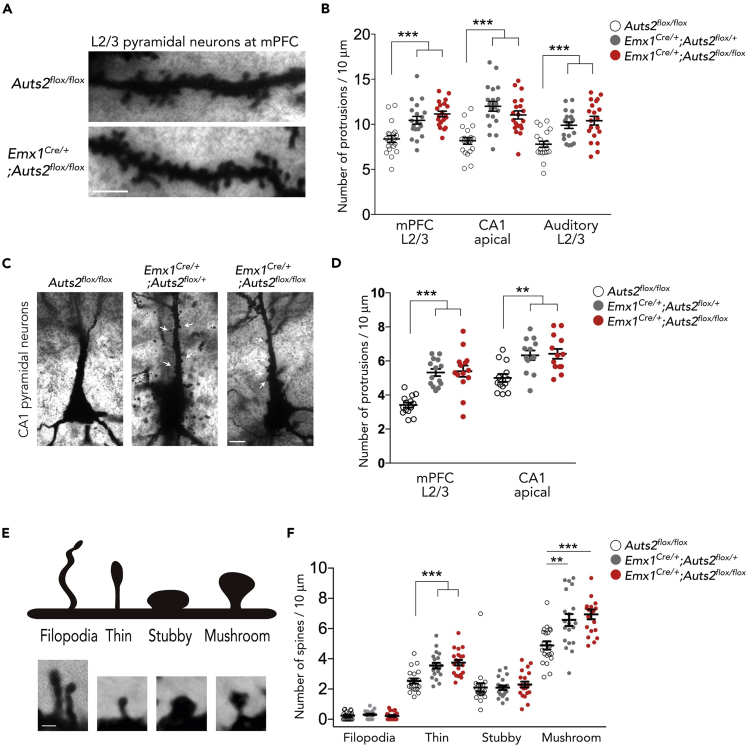
Loss of *Auts2* Abnormally Increases Dendritic Spine Formation *In Vivo* (A) Representative images of spines on the secondary dendritic segments from Golgi-stained upper-layer pyramidal neurons in the mPFC of control (*Auts2*^*flox/flox*^, upper panel) and *Emx1*^*Cre/+*^*;Auts2*^*flox/flox*^ homozygous mutant mouse brains (lower panel) at P35. (B) Summary graph of the spine density on the neurons at indicated areas in the control (*Auts2*^*flox/flox*^), heterozygous (*Emx1*^*Cre/+*^*;Auts2*^*flox/+*^), and homozygous (*Emx1*^*Cre/+*^*;Auts2*^*flox/flox*^) mutant mouse brains (n = 20 dendrites from n = 3 animals). (C) The representative images of Golgi-stained CA1 hippocampal pyramidal neurons in *Auts2*^*flox/flox*^, *Emx1*^*Cre/+*^*;Auts2*^*flox/+*^ heterozygotes, and *Emx1*^*Cre/+*^*;Auts2*^*flox/flox*^ homozygotes at P35. *Auts2* mutant neurons exhibited increased spines at apical primary dendrites (white arrows). (D) Summary graph of the spine density at apical primary dendrites on neurons in control (*Auts2*^*flox/flox*^), heterozygous (*Emx1*^*Cre/+*^*;Auts2*^*flox/+*^), and homozygous (*Emx1*^*Cre/+*^*;Auts2*^*flox/flox*^) mutant mouse brains (n = 13–15 dendrites from n = 3 animals). (E) Morphological classification of dendritic spines and filopodia. (F) The density of each category of spines in the upper-layer neurons in the mPFC was measured in the control (*Auts2*^*flox/flox*^), heterozygous (*Emx1*^*Cre/+*^*;Auts2*^*flox/+*^), and homozygous (*Emx1*^*Cre/+*^*;Auts2*^*flox/flox*^) mutant mouse brains (n = 20 dendrites from n = 3 animals). Data are presented as mean ± SEM. ∗∗p < 0.01, ∗∗∗p *<* 0.001, one-way ANOVA with Dunnett's post hoc test. Scale bar, 10 μm (A and C) and 2 μm (B).

Spine number generally increases with distance from the cell body in wild-type animals (Ballesteros-Yanez et al., 2006). We examined spine distribution along the middle dendritic segments of pyramidal neurons at multiple forebrain regions in adult brains. We found that spines were increased on the secondary dendrites of layer II/III pyramidal neurons in the medial prefrontal cortex (mPFC) in *Emx1*^*Cre/+*^*;Auts2*^*flox/flox*^ homozygous mutant brains compared with the *Auts2*^*flox/flox*^ controls (one-way ANOVA, p < 0.001, *F*_(2,57)_ = 14.67; Dunnett's post hoc test, ∗∗∗p < 0.001, Figures 2A and 2B). Significant differences were not limited to mPFC neurons. For example, increased spines were also observed on secondary dendritic segments along apical dendrites of hippocampal CA1 pyramidal neurons and dendrites of the upper-layer neurons of the auditory cortex (Figure 2B: one-way ANOVA, p < 0.001, *F*_(2,57)_ = 19.02; Dunnett's post hoc test, ∗∗∗p < 0.001 for CA1 apical and Auditory L2/3). Furthermore, we also observed analogous aberrant spine formation along primary apical dendrites immediately proximal to the cell soma of CA1 hippocampal pyramidal neurons as well as cortical layer II/III neurons in the *Auts2* mutant brains, a location where few spines were normally formed in wild-type animals (Figures 2C and 2D: Cortical layer II/III neurons, one-way ANOVA, p < 0.001, *F*_(2, 39)_ = 21.58; Dunnett's post hoc test, ∗∗∗p < 0.001 control versus Het or Homo. CA1 neurons: p < 0.001, *F*_(2, 36)_ = 8.719; Dunnett's post hoc test, ∗∗p = 0.002 control versus Het, ∗∗p = 0.001 control versus Homo). Interestingly, however, spine densities on basal dendrites of CA1 pyramidal neurons as well as both apical and basal dendrites of cortical layer 5/6 neurons at mPFC and auditory cortex were normal in *Auts2* mutant mice (Figure S3D: p = 0.800 for CA1 basal, p = 0.968 for mPFC L5/6 apical, p = 0.923 for mPFC L5/6 basal and p = 0.923 for auditory L5/6 basal). These findings suggest that AUTS2 restricts the number of dendritic spines within distinct dendritic compartments in selected neuronal populations. A similar phenotype was observed in *Emx1*^*Cre/+*^*;Auts2*^*flox/+*^ heterozygous mutants (∗∗∗p < 0.001, Figure 2B). Furthermore, *Auts2*^*del8/+*^ heterozygotes at adolescent (P30) as well as adult (P90) stages also displayed an increase in the densities of spines on the dendrites of both cortical and hippocampal CA1 pyramidal neurons compared with WT mouse brains (Figures S4A and S4B: ∗∗∗p < 0.001 for mPFC and CA1 neurons at P30, ∗∗p = 0.004 for mPFC neurons at P90, ∗∗∗p < 0.001 for CA1 neurons at P90).

Consistent with the increase of spines, immunohistochemical analysis revealed that the excitatory presynaptic marker VGLUT1 but not inhibitory VGAT-labeled puncta at mPFC was increased in *Auts2* mutant brains compared with the control mice, suggesting that loss of *Auts2* leads to an imbalance of excitatory and inhibitory synapse density (Figure S5: ∗∗∗p < 0.001 for VGLUT1, p = 0.070 for VGAT).

We categorized spines into four morphological types (Figure 2E) and found that both mature “mushroom” spines and immature “thin” spines were increased to a similar extent in dendrites of *Emx1*^*Cre/+*^*;Auts2*^*flox/+*^ heterozygous and *Emx1*^*Cre/+*^*;Auts2*^*flox/flox*^ homozygous or *Auts2*^*del8/+*^ mice (Figures 2F and S4C: Thin spine, one-way ANOVA, p < 0.001, *F*_(2,57)_ = 12.87; Dunnett's post hoc test, ∗∗∗p < 0.001 control versus Het or Homo. Mushroom spine, one-way ANOVA, p < 0.001, *F*_(2,57)_ = 10.67; Dunnett's post hoc test, ∗∗∗p = 0.002 control versus Het, ∗∗∗p < 0.001 control versus Homo). This indicates that AUTS2 does not affect the maturity of spines, as was also observed in our *ex vivo* data (Figures S2B–S2D). These observations suggest that AUTS2 restricts the number of excitatory synapses and that loss of one allele is sufficient to result in excessive excitatory synapses.

### *Auts2* Deficiency Causes Aberrant Excitatory Neurotransmission

**Figure 3:**
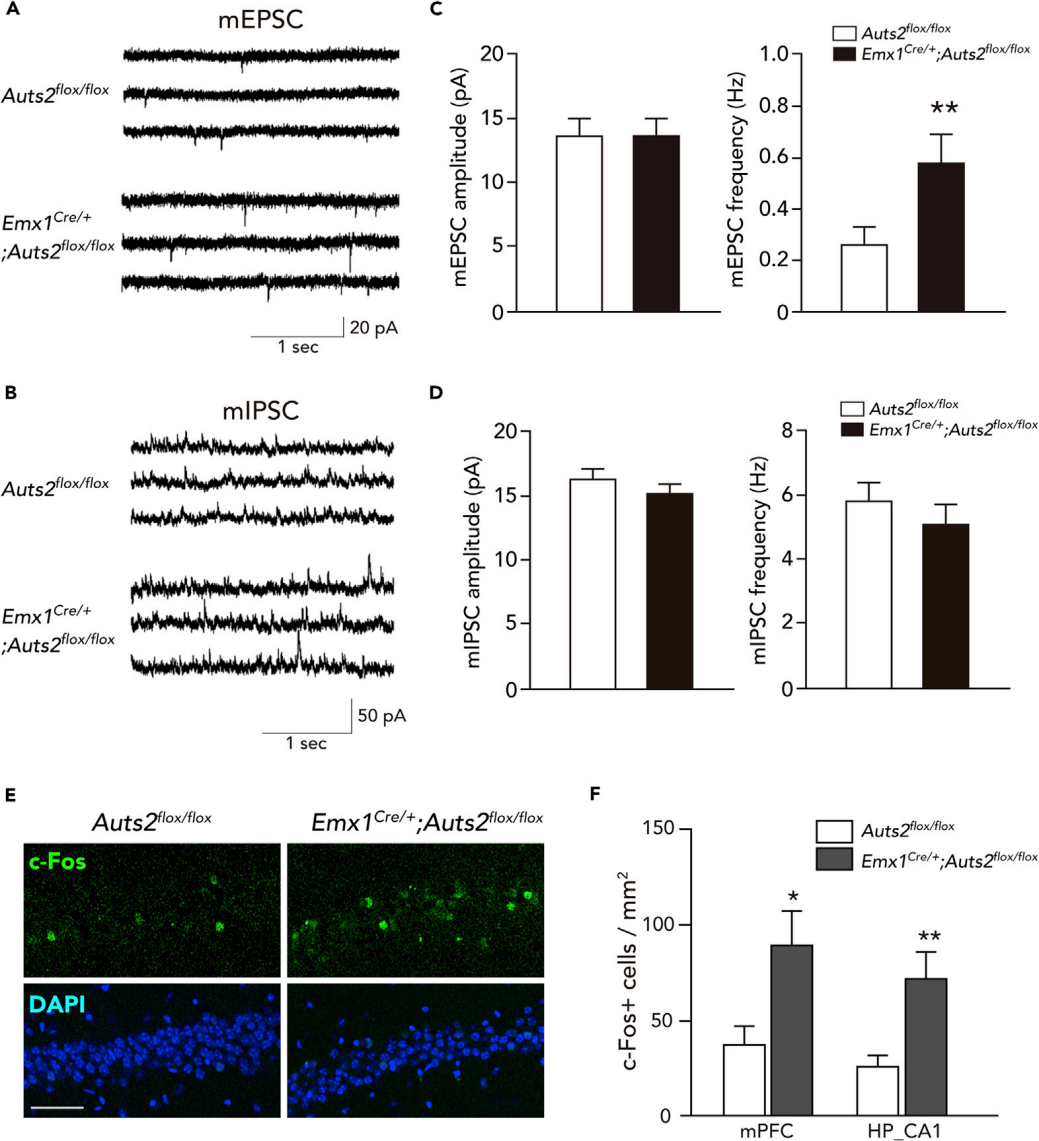
*Auts2* Mutant Mice Display Altered Synaptic Properties and Increased c-Fos Expression (A and B) Representative traces of mEPSCs (A) and mIPSCs (B) from slice recordings of CA1 pyramidal neurons from control (*Auts2*^*flox/flox*^) and *Emx1*^*Cre/+*^*;Auts2*^*flox/flox*^ homozygous mutant mice at P35. (C and D) *Emx1*^*Cre/+*^*;Auts2*^*flox/flox*^ mice exhibit increased mEPSC (C) but not mIPSC (D) frequencies without change in amplitude. n = 18–19 neurons from N = 6–8 mice per genotype. (E) Representative images of c-Fos expression in the hippocampal CA1 areas of homozygous *Emx1*^*Cre/+*^*;Auts2*^*flox/flox*^ homozygous mutant mice and *Auts2*^*flox/flox*^ control littermates. (F) Summary graphs of c-Fos-expressing cells in the indicated areas. About 8–12 tissue sections from N = 3 brains were analyzed. Data are presented as mean ± SEM. ∗p < 0.05, ∗∗p < 0.01, Mann-Whitney U test. Scale bar, 50 μm.

Next, we investigated the effect of *Auts2* inactivation on synaptic transmission properties. To address this, we performed whole-cell patch clamp recording of spontaneous miniature excitatory and inhibitory postsynaptic currents (mEPSCs and mIPSCs, respectively) on CA1 pyramidal neurons in acute hippocampal slices from P33–44 mouse brains. In the *Emx1*^*Cre/+*^*;Auts2*^*flox/flox*^ homozygous brains, the mEPSCs were increased in frequency (∗∗p = 0.006), in agreement with increased spines (Figures 3A and 3C). Furthermore, the average paired-pulse ratio of evoked EPSCs in response to paired sets of local stimulation was unchanged across the genotypes (p = 0.520, Figure S6), suggesting that the increase in mEPSC frequency observed in *Auts2* mutant brains is probably due to an increase in the number of functional excitatory synapses rather than an increase in the probability of presynapse release. On the other hand, the mEPSC in amplitude was unaltered (p = 0.954) compared with the control (*Auts2*^*flox/flox*^) mice (Figures 3A and 3C), suggesting that ablation of *Auts2* does not further promote the maturation of excitatory synapses. We also observed no significant difference in the mIPSCs with regard to either amplitude or frequency between the control and *Emx1*^*Cre/+*^*;Auts2*^*flox/flox*^ mutants (Figures 3B and 3D: p = 0.171 for amplitude, p = 0.252 for frequency).

Furthermore, we examined the expression of the immediate-early gene product, c-Fos, as a marker of neuronal activity in the brain (Sagar et al., 1988). Compared with the control (*Auts2*^*flox/flox*^) mice, a larger number of pyramidal neurons with strong c-Fos immunoreactivity were observed in the mPFC and hippocampal CA1 in *Emx1*^*Cre/+*^*;Auts2*^*flox/flox*^ homozygous mutants (Figures 3E and 3F: ∗p = 0.023 for mPFC, ∗∗p = 0.009 for CA1). This suggests that the disturbed balance between excitatory and inhibitory synaptic inputs in local neural circuits results in increased excitability in the *Auts2* mutant brains.

### *Auts2* Prevents Excessive Spine Formation Even after Developmental Stages

**Figure 4:**
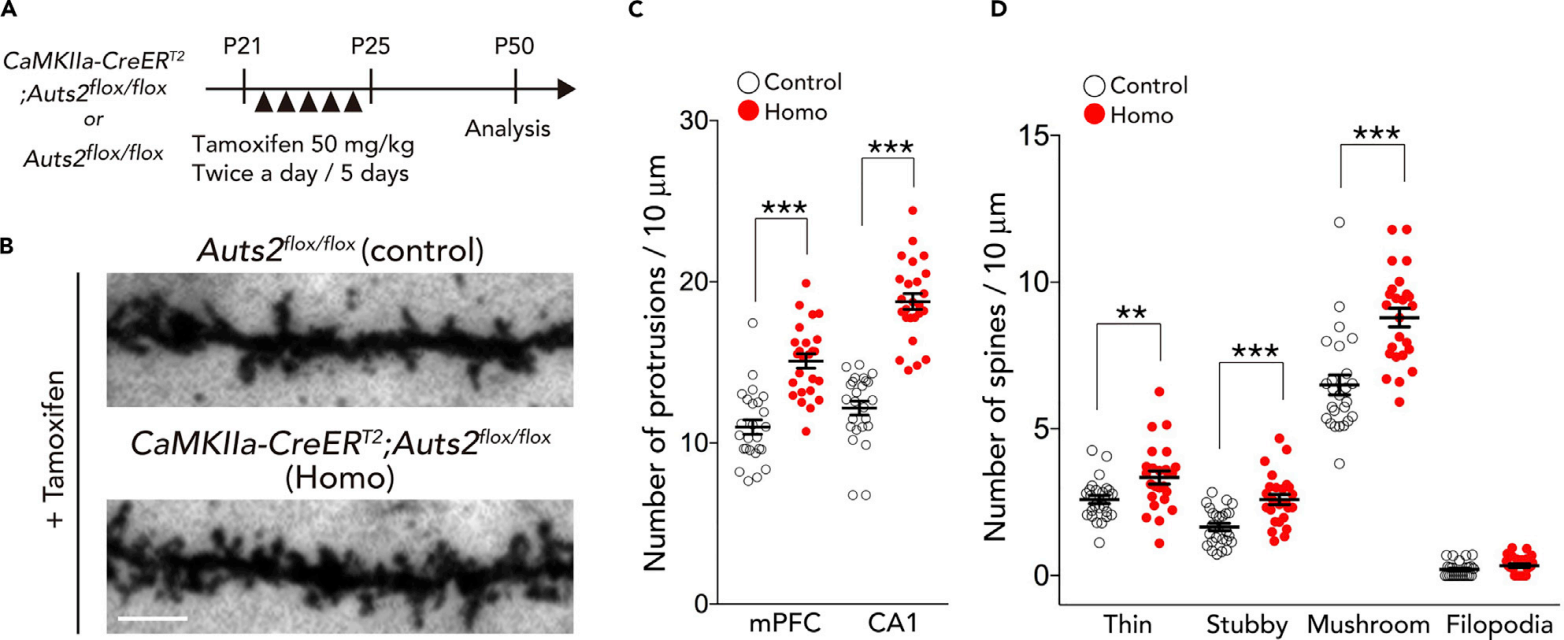
Conditional Deletion of *Auts2* in Postnatal Forebrain Leads to Excessive Spine Formation (A) Scheme illustrating the tamoxifen-inducible deletion of *Auts2* in postnatal forebrain. Tamoxifen was administered to *CaMKIIa-CreER*^*T2*^*;Auts2*^*flox/flox*^ homozygotes and their control *Auts2*^*flox/flox*^ littermate mice during P21–25, and analysis was performed at P50. (B) Representative images of the dendritic spines from Golgi-stained upper-layer pyramidal neurons at mPFC of the tamoxifen-treated control (*Auts2*^*flox/flox*^, upper panel) and *Auts2* homozygous mutant mouse brains (*CaMKIIa-CreER*^*T2*^*;Auts2*^*flox/flox*^, lower panel) at P50. (C) The pyramidal neurons in the mPFC as well as hippocampal CA1 area from mice postnatally lacking *CaMKIIa-CreER*^*T2*^*;Auts2*^*flox/flox*^ (Homo) exhibited increase of dendritic spines on the secondary dendritic segments relative to the *Auts2*^*flox/flox*^ littermates (control) (n = 25 dendrites from N = 3 animals). (D) The density of each category of spines on the pyramidal neurons in the mPFC was measured in control (*Auts2*^*flox/flox*^) and homozygous *CaMKIIa-CreER*^*T2*^*;Auts2*^*flox/flox*^ mutant mouse brains (n = 25 dendrites from N = 3 animals). Data are presented as mean ± SEM. ∗∗p < 0.01, ∗∗∗p < 0.001, unpaired t test. Scale bar, 10 μm.

Although our *ex vivo* and *in vivo* analyses suggest that AUTS2 regulates excitatory synapse formation, it is unclear whether AUTS2 possesses such a function after establishment of brain structures. To assess this issue, we crossed *Auts2-floxed* mice with *CaMKIIa-CreER*^*T2*^ mice to generate *CaMKIIa-CreER*^*T2*^*;Auts2*^*flox*^ mutant mice, in which the exon 8 of *Auts2* can be ablated in the forebrain projection neurons by administration of tamoxifen (Erdmann et al., 2007) (Figure S7A and Table S1). We have previously demonstrated that *Auts2* mutant mice displayed defects in neural development including neuronal migration and neurite outgrowth in a gene-dosage dependent manner (Hori et al., 2014, *Cell Rep*). Interestingly, however, the *Emx1*^*Cre/+*^*;Auts2*^*flox/+*^ heterozygous mutants exhibited aberrant spine formation to the same extent as the homozygotes (Figure 2B: p = 0.394 Het (*Emx1*^*Cre/+*^*;Auts2*^*flox/+*^) versus Homo (*Emx1*^*Cre/+*^*;Auts2*^*flox/flox*^) for mPFC; p = 0.305 Het versus Homo for CA1; p = 0.631 Het versus Homo for CA1, one-way ANOVA with Bonferroni post hoc test). To better understand the contribution of AUTS2 in postnatal synapse development as well as the *Auts2* phenotypes on mouse behaviors as described below, we examined *CaMKIIa-CreER*^*T2*^*;Auts2*^*flox/flox*^ homozygotes and *Auts2*^*flox/flox*^ control mice (Figures 4 and S11).

Tang et al. previously demonstrated that the *CaMKIIa*-promoter is active in forebrain neurons from postnatal week 3 to adulthood (Tang et al., 2014). When tamoxifen was administered during P21–25 to *CaMKIIa-CreER*^*T2*^*;Auts2*^*flox/flox*^ mutant mice and their control littermates (*Auts2*^*flox/flox*^), genomic recombination was detected in the mPFC and hippocampus but not in the cerebellum of *CaMKIIa-CreER*^*T2*^*;Auts2*^*flox/flox*^ mice (Figure S7B), indicating that this protocol efficiently induces the forebrain-specific Cre-mediated recombination. Induction of recombination was also confirmed by using *Rosa26R*^*YFP*^, a reporter allele to detect Cre-dependent recombination (Figure S7C). Quantitative RT-PCR revealed that *Auts2* mRNA levels dramatically decreased in the mPFC and hippocampus but not in the cerebellum of the tamoxifen-treated *CaMKIIa-CreER*^*T2*^*;Auts2*^*flox/flox*^ mice (Figure S7D: ∗∗∗p < 0.001 for mPFC, ∗∗p = 0.001 for HP, p = 0.054 for Cb).

Three weeks after tamoxifen administration to *CaMKIIa-CreER*^*T2*^*;Auts2*^*flox/flox*^ homozygous mutants and *Auts2*^*flox/flox*^ control mice (Figure 4A), *CaMKIIa-CreER*^*T2*^*;Auts2*^*flox/flox*^ mice displayed an increase in the densities of spines on the dendrites of both cortical and hippocampal pyramidal neurons (Figures 4B and 4C: ∗∗∗p < 0.001 for mPFC neurons, ∗∗∗p < 0.001 for CA1 neurons). Similar to the *Emx1*^*Cre/+*^*;Auts2*^*flox/flox*^ mutant mice, those increased spines consisted of mushroom and stubby-type mature spines as well as immature thin spines (Figure 4D: ∗∗p = 0.007 for thin spine, ∗∗∗p < 0.001 for stubby spine, ∗∗∗p < 0.001 for mushroom spine, p = 0.098 for filopodia). These findings suggest that AUTS2 is required for the dendritic spine number restriction even at post-developmental stages, which may contribute to the regulation of synaptic homeostasis.

### Aberrant Gene Expression in *Auts2* Mutant Mice

**Figure 5:**
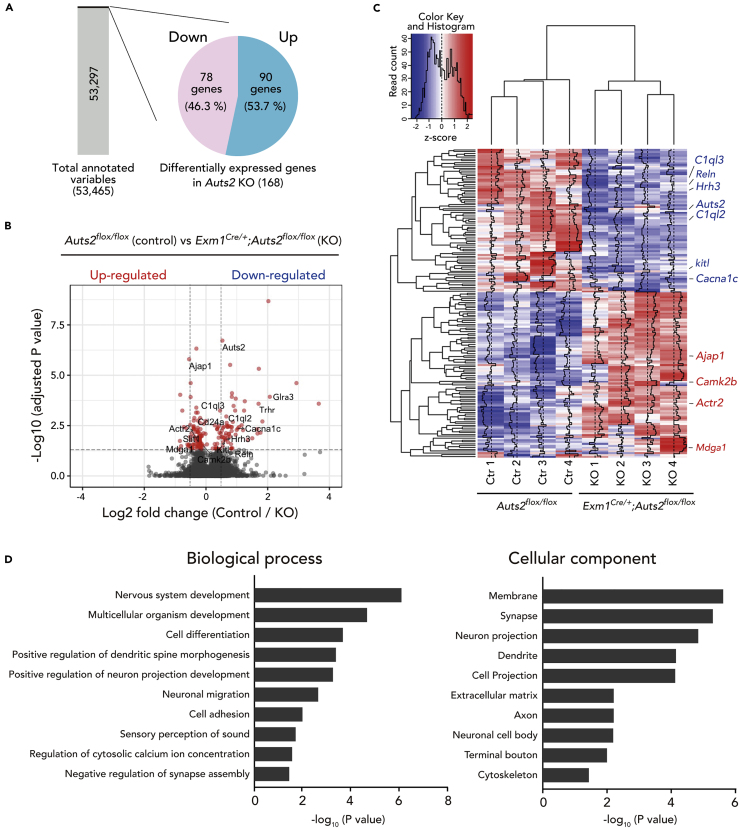
Transcriptome Analysis of *Emx1*^*Cre/+*^*;Auts2*^*flox/flox*^ Mutant Mice Hippocampal Brain Tissues Global gene expression analysis by RNA-sequencing reveals dysregulation of multiple genes associated with neurodevelopment. RNA samples from P14 hippocampus of *Emx1*^*Cre/+*^*;Auts2*^*flox/flox*^ homozygous mutant mice and the *Auts2*^*flox/flox*^ control littermates were used. (A) Rates in differentially expressed genes in *Emx1*^*Cre/+*^*;Auts2*^*flox/flox*^ homozygous mutant hippocampal tissues compared with the *Auts2*^*flox/flox*^ control littermates. (B) Volcano plot showing differential expression of all genes between *Auts2*^*flox/flox*^ (control) and *Emx1*^*Cre/+*^*;Auts2*^*flox/flox*^ homozygous mutants (KO). A threshold of 0.05 for the false discovery rate (FDR) and of 0.5 for log_2_ fold change (log2FC) were indicated by horizontal and vertical dashed lines, respectively. (C) Clustered heatmap of transcriptome analysis in *Emx1*^*Cre/+*^*;Auts2*^*flox/flox*^ homozygous mutants (KO) and the *Auts2*^*flox/flox*^ control littermates (Ctr). Four biological samples as indicated were subjected to RNA-seq analysis. Heatmap was generated by *Z* score calculated with the processed FPKM values for each differentially expressed gene. (D) Gene ontology (GO) analysis of the differentially expressed genes in *Auts2* mutant hippocampus.

The *ex vivo* rescue experiments in Figure 1E showed that AUTS2 in the nucleus functions to restrict the spine number. A previous study clarified that nucleic AUTS2 works as a component of PRC1 to participate in gene transcription (Gao et al., 2014). These findings suggest that AUTS2 protein in nuclei restricts spine formation by regulating gene expression of relevant neural genes. Therefore, we examined global mRNA expression profiles for *Emx1*^*Cre/+*^*;Auts2*^*flox/flox*^ homozygous brains and *Auts2*^*flox/flox*^ control littermate brains. In the postnatal mouse brains, the expression of AUTS2 in the cerebral cortex is downregulated to considerably lower levels and is confined to the prefrontal regions (Bedogni et al., 2010). In addition, the disturbed spine formation elicited by the ablation of *Atus2* is specific to the upper-layer neurons in the cerebral cortex (Figures 2B and S3D). In contrast, the hippocampus entirely sustains a higher level of AUTS2 expression even in mature brains. Thus, we prepared the RNA samples from the hippocampi of 2-week-old *Auts2* homozygous mutants and the control littermates for RNA sequencing (RNA-seq) analysis. Through RNA-seq, we identified a total of 168 genes, whose expression levels were significantly altered (false discovery rate [FDR] < 0.05) in the mutant hippocampus, with 78 downregulated and 90 upregulated genes expressed as Log_2_FKPM (fragments per kilobase of exon per million reads mapped) (Figures 5A–5C and Data S1). Interestingly, these differentially expressed genes included the genes encoded synaptic proteins or molecules involved in synaptic functions, such as *Reln*, *Mdga1*, *Camk2b*, *Cacna1c*, and *C1ql*-family genes (Fink et al., 2003, Gangwar et al., 2017, Martinelli et al., 2016, Matsuda et al., 2016, Moosmang et al., 2005, Pettem et al., 2013, van Woerden et al., 2009, Wasser and Herz, 2017) (Figures 5B and 5C). Gene ontology (GO) analysis revealed that these altered genes were associated with multiple aspects of neurodevelopment including “nervous system development,” “cell differentiation,” and “neuronal migration,” with particular enrichment of the terms for synapse development such as “dendritic spine morphogenesis,” “negative regulation of synapse assembly,” and “regulation of cytosolic calcium ion concentration” (Figure 5D and Data S2). Among the genes categorized in GO cellular components such as “Membrane” or “Synapse,” six up-regulated (e.g., *Mdga1, Camk2b*, and *sema6b*) and thirteen down-regulated genes (e.g., *Dcc, Gfra1, Gpc2, Hap1*) overlapped with genes categorized in the biological process “nervous system development” (Figure S8). These results suggest that nucleic AUTS2 regulates the expression of genes that are related to synapse formation/function and some of which may be involved in spine number restriction. Aberrant expression of such synaptic genes may cause synaptic dysfunction in patients with *AUTS2* mutations.

### Loss of *Auts2* Impairs Social Behaviors

In our previous studies, the heterozygotic mouse mutants for another *Auts2* allele, *Auts2*^*neo/+*^, whose AUTS2 expression profile is distinct from that of *Auts2*^*del8/+*^ (Table S1), displayed the behavioral abnormalities in cognition and emotional control while behaving normally in social interaction (Hori et al., 2014, Hori et al., 2015). Human genetic studies have previously reported that individuals with mutations in *AUTS2* locus exhibited common features including ID, developmental delay, microcephaly, and epilepsy but distinct psychiatric disorders such as ASDs, ADHD, and schizophrenia (Oksenberg and Ahituv, 2013). One plausible hypothesis is that the heterogeneity of structural variants in the *AUTS2* locus could result in the expression of phenotypic variation between the patients with *AUTS2* mutations. This prompted us to examine the social behaviors of *Auts2*^*del8/+*^ mice, especially focusing on mouse social communications.

All experimental mice including *Auts2*^*del8/+*^ mutants, tamoxifen-treated *CaMKIIa-CreER*^*T2*^*;Auts2*^*flox/flox*^ mice, and *Auts2*^*flox/flox*^ control littermates appeared grossly normal. All of them had normal fur and whiskers and showed no detectable motor disability. The body weight of *Auts2*^*del8/+*^ mice was slightly decreased compared with WT littermates (body weight at 3 months of age; WT, 27.94 ± 0.54 g [n = 16]; *Auts2*^*del8/+*^, 20.50 ± 0.35 [n = 16]; data are mean ± SEM, Mann-Whitney *U* = 2.5, ∗∗∗p < 0.001).

**Figure 6:**
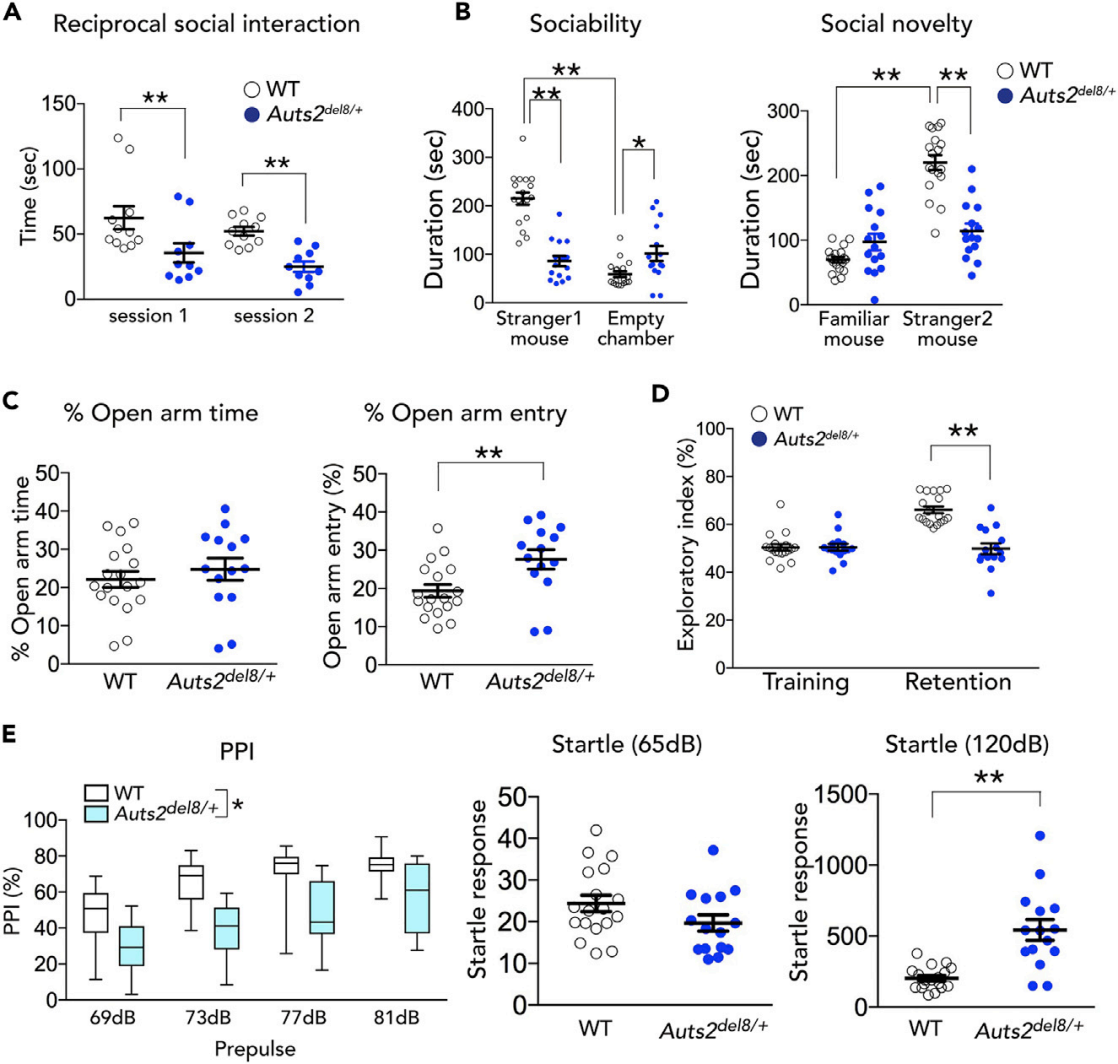
Behavioral Abnormalities in *Auts2*^*del8/+*^ Mutant Mice (A) Reciprocal social interaction test. Social interaction between WT or *Auts2*^*del8/+*^ mouse pairs during 5 min were measured (WT, n = 11, *Auts2*^*del8/+*^, n = 10). (B) Three-chamber social interaction test. Graphs show the amount of time spent in each chamber (WT, n = 18, *Auts2*^*del8/+*^, n = 15). (C) *Auts2*^*del8/+*^ mice exhibit increased open arm entry relative to WT mice in elevated plus maze test (WT, n = 18, *Auts2*^*del8/+*^, n = 14). (D) *Auts2*^*del8/+*^ mice display deficits in novel object recognition. Graphs show the exploratory preference in training and retention sessions (WT, n = 18, *Auts2*^*del8/+*^, n = 15). (E) Prepulse inhibition (PPI) (%) at four different prepulse intensities in PPI test (left graph) and acoustic startle response (middle and right graphs) as measured in trials without a prepulse. *Auts2*^*del8/+*^ mice display decrease of the percentage of PPI as well as a higher acoustic startle response at 120 dB pulse relative to those in WT mice (WT, n = 18, *Auts2*^*del8/+*^, n = 15). Data are mean ± SEM and box-and-whisker plots (medians with interquartile range, minimum, and maximum values are represented). ∗p < 0.05, ∗∗p < 0.01,; (A and B) two-way ANOVA, (C) unpaired t test, (D) two-way ANOVA with repeated measures, (E) two-way ANOVA with repeated measures in PPI test and Mann-Whitney U-test in startle response.

We performed the reciprocal dyadic social interaction test to evaluate social behavior, in which mice were allowed to freely move and reciprocally interact with each other (Harper et al., 2012, Hiramoto et al., 2011). *Auts2*^*del8/+*^ mice displayed lower levels of active affiliative social interaction than WT mice in both session 1 and session 2 (Figure 6A: ∗∗p = 0.001 for session 1, ∗∗p = 0.009 for session 2). Of note, the restricted ablation of *Auts2* in mature excitatory neurons in the adult forebrain well recapitulated the impairment of social interaction, as depicted by tamoxifen-treated *CaMKIIa-CreER*^*T2*^*;Auts2*^*flox/flox*^ mutants (Figures S11A and S11D: ∗∗p = 0.001 for session 1, ∗p = 0.038 for session 2). Furthermore, in a three-chamber social interaction test, *Auts2*^*del8/+*^ mutant mice displayed a decreased preference for a social subject (stranger mice 1 and 2) over non-social subject (empty chamber or familiar mouse) compared with WT mice in both sociability and social novelty phases (Figure 6B). These results suggest that *Auts2* mutant mice have social defects. We confirmed that sensory abilities such as olfaction and visual functioning as well as tactile response were not significantly different across the genotypes, as no phenotype was observed in the buried food finding test (Figure S9A: p = 0.065; Figure S11C: p = 0.707), whisker twitch reflex (100% response in WT, n = 12, *Auts2*^*del8/+*^, n = 10, *Auts2*^*flox/flox*^, n = 10 and *CaMKIIa-CreER*^*T2*^*;Auts2*^*flox/flox*^, n = 10), and visual placing response test (p = 0.898, Figure S9B; p = 0.557, Figure S11B), respectively. To further examine the sensory function of the vibrissae, we measured thigmotactic behaviors, defined as movement along the walls so that one side of the vibrissae could contact and scan the edge of the wall (Luhmann et al., 2005, Milani et al., 1989). *Auts2*^*del8/+*^ mutant and WT mice behaved similarly in this test (Figure S9C: time × genotype interaction, *F*_(3,54)_ = 0.337, p = 0.799; genotype, *F*_(1,18)_ = 0.670, p = 0.424; time, *F*_(3,54)_ = 4.06, p = 0.011). These results suggest that the impaired social interaction probably does not involve the alterations in non-specific elements of social behavior such as sensory functioning.

### Other Behavioral Phenotypes of *Auts2*^*del8/+*^ Mice

Spontaneous locomotor activity test showed that the *Auts2*^*del8/+*^ mice exhibited significantly decreased exploratory behavior during the first 15 min of the test (Figure S10A: time × genotype interaction, *F*_(2,66)_ = 7.61, p = 0.001; genotype, *F*_(1,33)_ = 21.68, p < 0.001; time, *F*_(2,66)_ = 5.07, p = 0.009).

In the open field test, the time that *Auts2*^*del8/+*^ mice spent in the illuminated inner area was comparable with that of WT mice, although general locomotor activity was slightly reduced in *Auts2*^*del8/+*^ mice as indicated by total travel distance during the test (Figure S10B: time spent in inner sector, p = 0.697; total distance traveled, ∗∗∗p < 0.001). In the elevated plus maze test, however, *Auts2*^*del8/+*^ mice displayed increased exploratory behavior of the open arms compared with WT mice, suggesting that *Auts2*^*del8/+*^ mice have reduced fear of height (∗∗p = 0.008, Figure 6C).

In a novel object recognition test, *Auts2*^*del8/+*^ mice exhibited impaired recognition memory performance depicted by the significant decrease of time for exploratory index to the novel object (Figure 6D: session × genotype interaction, *F*_(1,62)_ = 25.63, p < 0.001; genotype, *F*_(1,62)_ = 25.15, p = 0.001; session, *F*_(1,62)_ = 21.74, p < 0.001). Meanwhile, *Auts2*^*del8/+*^ mice showed normal associative memory functions in the fear-conditioning test (Figure S10C: context-dependent, p = 0.175; tone-dependent, p = 0.841). Interestingly, *Auts2*^*del8/+*^ exhibited a higher response to nociceptive stimuli as observed in the *Auts2*^*neo/+*^ mutants in our previous study (∗∗∗p < 0.001, Figure S10C) (Hori et al., 2015). Furthermore, *Auts2*^*del8/+*^ exhibited abnormal acoustic startle responses as well as sensorimotor gating deficits as indicated by decrease in the percentage of prepulse inhibition (Figure 6E: prepulse × genotype interaction, *F*_(3,93)_ = 3.31, p < 0.023; genotype, *F*_(1,31)_ = 19.77, p < 0.001; prepulse, *F*_(3,93)_ = 74.83, p < 0.001 for PPI; p = 0.103 for startle response to a 60 dB, *∗∗∗*p < 0.001 for startle response to a 120 dB).

### Altered Vocal Communication in *Auts2* Mutant Mice

**Figure 7:**
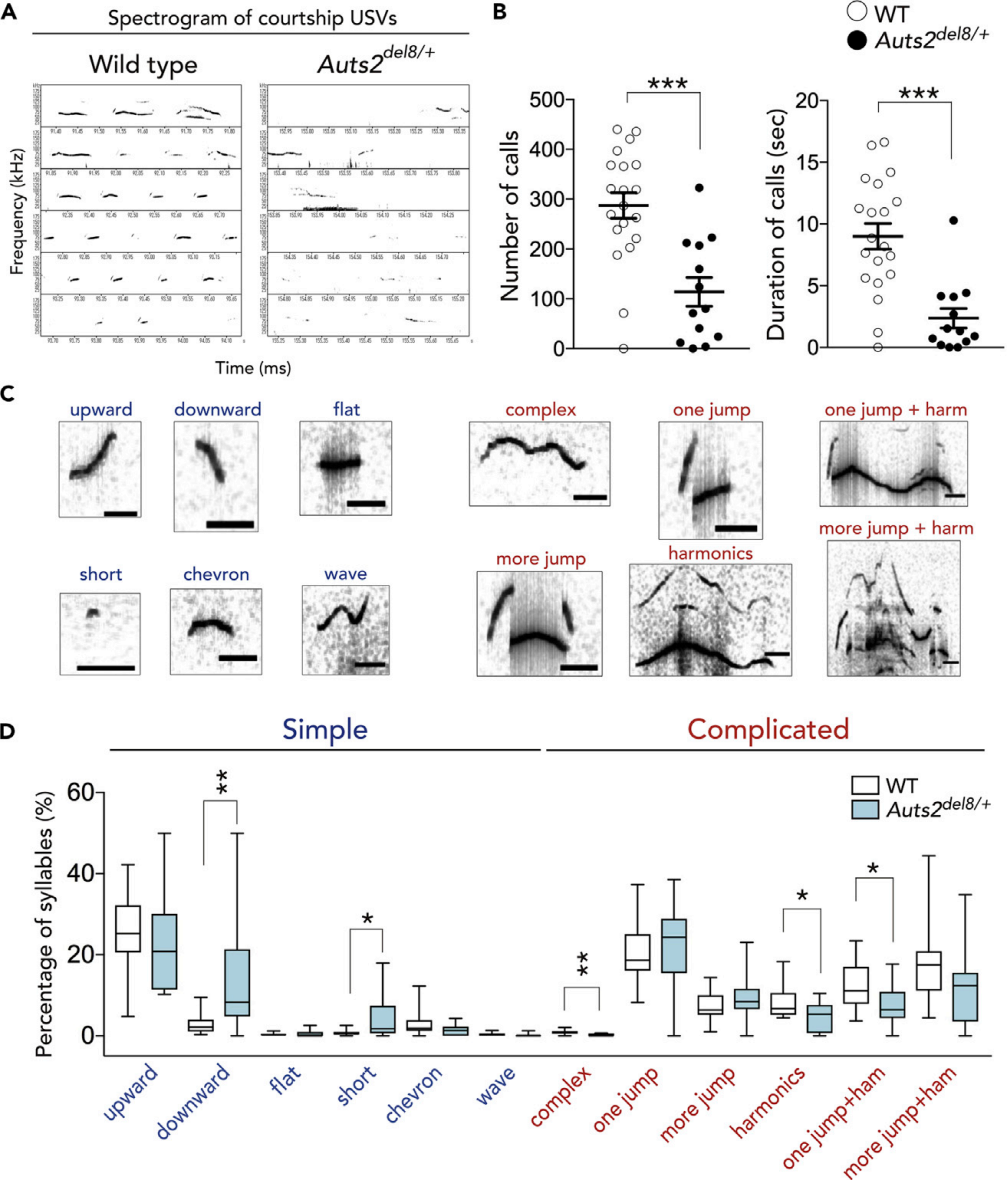
Deficits in Vocal Communication in Adult *Auts2*^*del8*^ Mutant Mice (A) Representative spectrograms of USV during the courtship behaviors. (B) The number (left) and duration (right) of USVs during 1 min. (C) Typical spectrograms of 12 different call patterns. Six simple call types (blue) and six complicated call types (red) are indicated. (D) The frequency of each syllable pattern is shown as the percentage of total calls. Data are mean ± SEM and box-and-whisker plots (medians with interquartile range, minimum, and maximum values are represented) (WT, n = 20, *Auts2*^*del8/+*^, n = 13). ∗p < 0.05, ∗∗p < 0.01, ∗∗∗p < 0.001; (B) unpaired t test, (D) Mann-Whitney U test.

Among types of social behaviors, mouse vocal communication has recently received attention as a possible model for studying the genetic and neural mechanisms for social communication (Holy and Guo, 2005). Mice use ultrasonic vocalizations (USVs) to exchange information in a variety of social contexts (Portfors and Perkel, 2014). When interacting with females, adult WT males actively emit courtship USVs with key tone frequencies between 50 and 80 kHz, as observed in the real-time spectrograms in Figure 7A. In contrast, the USVs produced by *Auts2*^*del8/+*^ males were apparently dispersive during the test (Figure 7A). Indeed, the mean number and duration of USVs were markedly reduced in *Auts2*^*del8/+*^ mice compared with WT controls (Figure 7B: ∗∗∗p < 0.001 for call number; ∗∗∗p < 0.001 for duration). Similarly, *CaMKIIa-CreER*^*T2*^*;Auts2*^*flox/flox*^ males also displayed the altered vocalizations (Figure S11E: ∗∗p = 0.003 for call number; p = 0.058 for duration). The experiments of auditory playback previously showed that adult females prefer USVs with greater complexity from neonates as well as adult males (Chabout et al., 2015, Takahashi et al., 2016). We classified the acoustic structures of USVs into 12 different call patterns and grouped them into “simple” and “complicated” syllable types (Figure 7C). *Auts2*^*del8/+*^ emitted significantly fewer numbers of the complicated syllable type, including “harmonics,” “complex,” or “one jump + harmonics,” whereas the simple syllable types with shorter duration such as “downward” or “short” were significantly increased (Figure 7D: ∗∗p = 0.002 for downward; ∗p = 0.025 for short; ∗∗p = 0.001 for complex; ∗p = 0.022 for harmonics; ∗p = 0.025 for one jump + harmonics). These findings suggest that loss of *Auts2* alters mouse vocal communication, which may underlie the pathology for communication disorders in patients with ASD with *AUTS2* mutations.

## Discussion

In this study, we found that AUTS2 restricts the number of excitatory synapses in forebrain pyramidal neurons, such as mPFC, and in the hippocampus, which are implicated as the critical regions for socio-communicative and cognitive brain functions. In *Auts2* mutant forebrains, the aberrant dendritic spine formation leads to the enhancement of excitatory synaptic inputs, which results in the changes in a balance between excitation and inhibition (E/I) that is observed in several otherwise different neuropsychiatric disorders such as ASDs and schizophrenia as well as mouse models (Lee et al., 2017, Penzes et al., 2011). These findings suggest a potential link between the behavioral abnormalities in *Auts2* mutant mice and the aberrant dendritic spine development.

Interestingly, in *Auts2* mutant cerebral cortex, aberrant spine formation specifically appeared in the upper-layer but not deep-layer neurons, although AUTS2 is widely expressed in both cortical layers (Figures 2B and S3D) (Bedogni et al., 2010). One plausible hypothesis is that AUTS2 may have distinct roles for neural development in different cerebral cortical areas, which may depend on differences of AUTS2 isoforms expressed between neurons or on co-factors that differentially interact with each AUTS2 isoform. Monderer-Rothkoff et al. have recently demonstrated that the long and short AUTS2 isoforms, each interacting with different co-factors, act opposingly on gene transcription in a cellular-context-dependent manner (Monderer-Rothkoff et al., 2019).

Electrophysiological experiments revealed that excitatory but not inhibitory synaptic inputs were elevated in the *Auts2* mutant hippocampal slices where strong c-Fos signals were observed, implying that the E/I balance was disturbed in that region. E/I balance in neural circuits is tightly controlled and established by contributions from a large number of factors in the normal brain. Accumulating evidence implicates a disturbed E/I balance within cortical neural circuitry in various neuropsychiatric disorders including ASD, anxiety, and ADHD (Chao et al., 2010, Edden et al., 2012, Gogolla et al., 2009, Han et al., 2012, Rubenstein and Merzenich, 2003). Although a recent report suggests that E/I imbalance is not causative for the neuropathology of the disorders but reflects a homeostatic response in some mouse models (Antoine et al., 2019), the hyperexcitability caused by an increased E/I ratio in the cerebral cortex is thought to be one potential common mechanism underlying the neurobehavioral defects of some forms of ASD via a distinct molecular pathway (Lee et al., 2017).

During the spinogenesis, a rapid increase of dendritic spine density occurs in the forebrain neurons, in which the gain of spines exceeds loss of spines, eventually causing excessive excitatory synapses for the formation of neural circuits (Chen et al., 2014, Forrest et al., 2018, Isshiki et al., 2014, Penzes et al., 2011). Thereafter, the growth of excitatory synapses is gradually downregulated and unnecessary spines are selectively pruned, after which spines are maintained during adulthood. Time-lapse imaging experiments using *Auts2*-knocked-down hippocampal neurons revealed that *de novo* formation of dendritic spines is promoted, whereas the elimination rate is decreased, resulting in the exaggerated formation of excitatory synapses. These observations suggest an important role for AUTS2 in controlling the number of spines or excitatory synapses in forebrain neurons by modulating their turnover. We found that this excess in synapses was also observed in tamoxifen-treated *CaMKIIa-CreERT2;Auts2*^*flox/flox*^ in which *Auts2* was ablated after establishment of the brain structure. This suggests that AUTS2 is involved in regulating synaptic homeostasis at late developmental and/or adult stages.

Emerging evidence indicates that aberrant regulation of spine number and/or an increased excitatory synaptic inputs likely caused by incomplete pruning or exaggerated formation of spines is associated with numerous pathological conditions such as ASD, schizophrenia, and neurodegenerative disorders (Chen et al., 2014, Forrest et al., 2018, Lee et al., 2017, Penzes et al., 2011). Transcriptional control by epigenetic regulation including histone post-translational modification and chromatin remodeling is critical in synapse development and neurological disorders. A recent study by Korb et al. revealed that Fragile X mental retardation protein *Fmr1* mutant mice exhibit widespread histone mis-modifications (Korb et al., 2017). These are associated with open chromatin caused by upregulation of epigenetic factor Brd4, resulting in alteration of the transcription levels of many critical synapse-related genes. In this study, we showed that nuclear-localizing AUTS2 functions restrict spine number. Because AUTS2 is involved in transcriptional regulation via chromatin modification as a component of PRC1 (Gao et al., 2014), and because expression of many synapse-related genes was altered in the *Auts2* mutants (Figure 5), we believe that nuclear AUTS2 restricts the excitatory synapse number via controlling the expression of relevant genes, thus maintaining the excitation/inhibition balance of the brain.

In previous and current studies, we characterized behavioral phenotypes for two lines of mutant mice with different mutations disrupting the *Auts2* locus (Hori et al., 2015). We summarized the results from a behavioral test battery for *Auts2*^*neo/+*^ (Hori et al., 2015) and *Auts2*^*del8/+*^ mutant mice (this study) in Figure S10D. In this study, we found that the *Auts2*^*del8/+*^ heterozygous global KO as well as *CaMKIIa-CreER*^*T2*^*;Auts2*^*flox/flox*^ conditional KO mice exhibited autistic-like behaviors including social deficits and altered vocal communications as well as multiple other behavioral impairments. In addition, *Auts2*^*del8/+*^ mice also showed altered anxiety as well as higher responses against nociceptive and auditory stimuli, both of which are often observed in patients with ASD (American Psychiatric Association, 2013). Interestingly, *Auts2*^*del8/+*^ mutant mice share several behavioral phenotypes with *Auts2*^*neo/+*^ mutants but also display a distinct combination of phenotypes (Figure S10D). Although the mechanisms underlying how different mutations lead to the distinct behavioral phenotypes in mice remains unclear, it is possible that compensatory expression of an AUTS2 C-terminal short isoform (S-AUTS2 var2) in *Auts2*^*del8/+*^ mutant brains negatively affects social behaviors in the social interaction tests (Figures 6A and 6B), whereas it alleviates the cognitive dysfunctions displayed in *Auts2*^*neo/+*^ mutant mice (Hori et al., 2015) such as the associative memory formation in fear-conditioning tests (Figure S10C). Alternatively, structural changes of the *Auts2* gene locus in these mutant mice could differentially impact on the expression of other AUTS2 isoforms, leading to the distinctive behavioral phenotypes, although we do not have a direct evidence of this. Further comparative analyses between these *Auts2* mutants will help us to understand the physiological function of AUTS2 in synapse development and the pathology of the *AUTS2*-related psychiatric illnesses.

In humans, it has been reported that multiple types of heterozygous genomic structural variants in the *AUTS2* locus including *de novo* balanced translocation, inversion, or intragenic deletions are associated with a wide range of psychiatric illnesses such as ASDs, ID, ADHD, schizophrenia, and dyslexia, as well as other neuropsychiatric diseases (Oksenberg and Ahituv, 2013). In addition to the exonic deletions of the *AUTS2* locus, some of the genomic structural variants are within non-coding regions including intronic and 5′ upstream regions, implying that improper and disorganized expression of AUTS2 could be involved in the onset of the disorders. However, it remains largely unclear how different mutations of the same gene contribute to different diseases. Currently, eight computationally annotated *AUTS2* isoforms in humans are incorporated in public databases (for example, the UCSC Genome Bioinformatics ([https://genome.ucsc.edu]). However, the study by Kondrychyn et al. revealed that *auts2a*, the zebrafish ortholog of *Auts2*, possesses 13 putative unique transcriptional start sites (TTS) and, surprisingly, more than 20 alternative transcripts are potentially produced from this gene locus by the aforementioned TSSs and/or by alternative splicing (Kondrychyn et al., 2017). These findings suggest that mammals including mouse and human could have similar or higher transcriptional complexity for *Auts2*/*AUTS2* than previously thought. Furthermore, Oksenberg et al. have identified several enhancer regions for the expression of *auts2a/Auts2* in zebrafish and mouse brain within the intronic regions of this gene locus (Oksenberg et al., 2013). Therefore, structural variants such as genomic deletions within a certain region of *Auts2/AUTS2* locus could not only alter the expression of full-length AUTS2 directly but also affect the transcriptional regulation of other AUTS2 isoforms. Different mutations of the *AUTS2* gene may differentially alter the temporal and spatial expression profiles of AUTS2 isoforms in various brain regions, which may distinctively affect neurobiological functions, ultimately resulting in the occurrence of multiple types of psychiatric disorders in individuals with AUTS2 syndrome. Our previous and this study, thus, highlighted that two types of *Auts2* mutants with different AUTS2 protein expression profiles exhibited overlapping but distinct behavioral abnormalities. This may support the notion that different types of mutations in *AUTS2* account for distinct types of neuropsychiatric illnesses. Future comprehensive studies elucidating the regulatory mechanisms for transcription/splicing of *Auts2/AUTS2* as well as neurobiological functions of the distinctive AUTS2 isoforms will help us to understand the pathogenic mechanisms underlying the occurrence of a variety of psychiatric disorders in individuals with *AUTS2* mutations and could contribute to therapeutic development for *AUTS2*-related neurological disorders.

In conclusion, the findings presented here suggest that synaptic regulation by AUTS2 is required for proper social behaviors. Furthermore, our results from the behavioral analyses for *Auts2*^*del/8/+*^ KO mice provided insight into the involvement of AUTS2 in other higher brain functions such as recognition and emotion. In addition to the AUTS2 function on synapse regulation, AUTS2 is also involved in neuronal migration and neurite formation (Hori et al., 2014). Therefore, the other abnormal behaviors observed in *Auts2*^*del/8/+*^ or *Auts2*^*neo/+*^ KO mice may partly be caused by the impairments in these developmental processes. Comparative analyses of the different forms of *Auts2* mouse mutants will help us to better understand the pathological mechanisms of the psychiatric disorders caused by *AUTS2* mutations. *Auts2* conditional KO mice with *CaMKIIa-CreER*^*T2*^ or other more restricted-expression forms of *Cre* will be useful for dissecting the distinct neural circuitries involved in these abnormal behaviors.

### Limitations of the Study

In this study, we demonstrated that the nuclear AUTS2 controls the number of excitatory synapses in the forebrain pyramidal neurons, possibly by regulating the expression of genes for synapse development and functions. Transcriptome analysis revealed that loss of *Auts2* alters the expression levels of multiple synapse-related genes as well as genes for neuronal morphogenesis. The current study, however, does not address the mechanisms underlying the regulation of AUTS2 in the expression of these synapse-related genes. Moreover, the AUTS2 downstream targets that are responsible for dendritic spine development remains to be determined. Electrophysiological experiments reveal that increased dendritic spines caused by *Auts2* ablation in mice leads to the enhancement of excitatory synaptic inputs, resulting in a disturbed balance in excitatory and inhibitory synaptic inputs. We have not, however, evaluated the effects on synaptic plasticity such as long-term potentiation/depression. Further studies are required to address these issues to obtain a more complete picture of synaptic pathology caused by *AUTS2* mutations.

### Resource Availability

#### Lead Contact

Further information and requests for resources should be directed to and will be fulfilled by the Lead Contact, Mikio Hoshino (hoshino@ncnp.go.jp).

#### Materials Availability

All unique materials generated from this study are available from the Lead Contact with a complete Materials Transfer Agreement.

#### Data and Code Availability

RNA-seq data have been deposited into GEO database with the accession number GSE134712.

## Methods

All methods can be found in the accompanying Transparent Methods supplemental file.

## Supplemental Information

Document S1. Transparent Methods, Figures S1–S11, and Table S1

Data S1. Differentially Expressed Gene List, Related to Figure 5

Data S2. GO Analysis Results, Related to Figure 5
