# Supplementary material for: AUTS2 Regulation of Synapses for Proper Synaptic Inputs and Social Communication
Source: iScience. 2020 May 18;23(6):101183. doi: 10.1016/j.isci.2020.101183 (PMC7267731; doi:10.1016/j.isci.2020.101183)
Supplement: Document S1. Transparent Methods, Figures S1–S11, and Table S1 [file mmc1.pdf]

iScience, Volume *23*

Supplemental Information

AUTS2 Regulation of Synapses for Proper Synaptic Inputs and Social Communication

Kei Hori, Kunihiko Yamashiro, Taku Nagai, Wei Shan, Saki F. Egusa, Kazumi Shimaoka, Hiroshi Kuniishi, Masayuki Sekiguchi, Yasuhiro Go, Shoji Tatsumoto, Mitsuyo Yamada, Reika Shiraishi, Kouta Kanno, Satoshi Miyashita, Asami Sakamoto, Manabu Abe, Kenji Sakimura, Masaki Sone, Kazuhiro Sohya, Hiroshi Kunugi, Keiji Wada, Mitsuhiko Yamada, Kiyofumi Yamada, and Mikio Hoshino

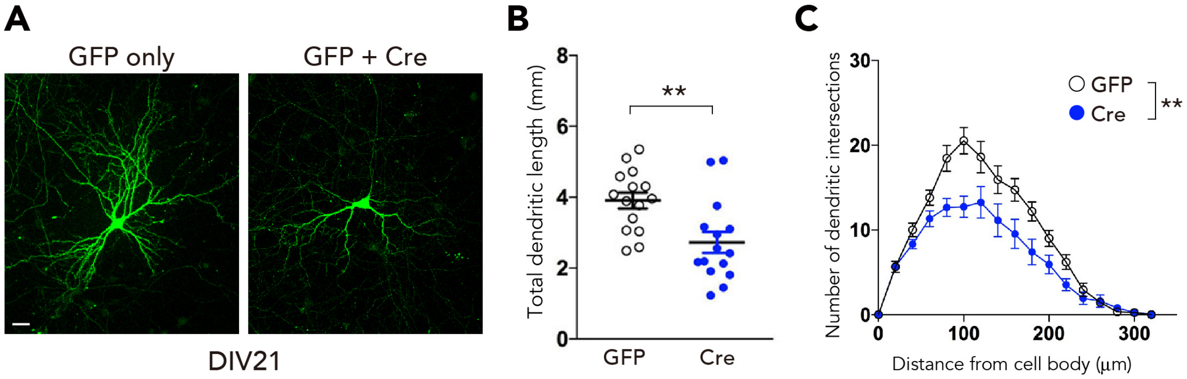

Figure S1. ***Auts2*** **mutant primary hippocampal neurons exhibit the impairment of dendritic morphogenesis, Related to Figure 1.**

(A) Representative images of cultured primary hippocampal control (GFP only) and *Auts2*-deficient neurons (GFP + Cre) at DIV21. Neurons derived from *Auts2*$^{flox/flox}$ homozygotic brains were electroporated with control or Cre expression vectors at DIV0. GFP expression vector was co-electroporated to visualize the neurons. (B) Measurement of total dendritic length (n = 15 neurons). (C) Sholl analysis. Graph shows the number of dendritic intersections of dendrites per each Sholl ring (20 μm interval concentric circles centered on the soma) for the control (GFP only) and *Auts2*-deficient (GFP + Cre) neurons at DIV21 (n=15 neurons). Data are mean ± SEM. $^{**}P < 0.01$. (B) unpaired t-test, (C) repeated-measure ANOVA. Scale bar, 20 μm.

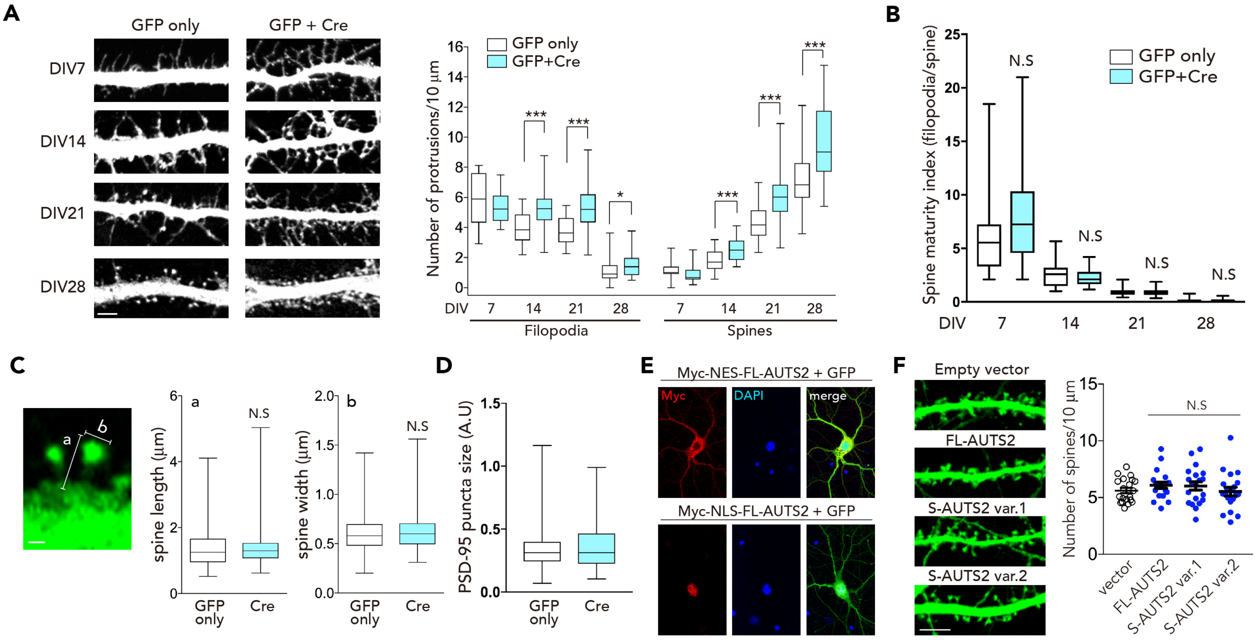

Figure S2. *Auts2* mutant primary neurons exhibit excessive spine formation, Related to Figure 1.

(A) The representative images of secondary dendritic segments of cultured primary hippocampal neurons. The neurons derived from *Auts2*$^{flox/flox}$ homozygotes were electroporated with the control or Cre expression vectors. To visualize the neurons, GFP expression vector was co-electroporated. The density of dendritic spines and filopodia on the dendrites of the control and *Auts2* KO (Cre) neurons were measured at different culture stages (DIV7-28) (n=21-40 dendrites of 11-20 neurons). (B) Graph shows the spine maturity index defined by the ratio of filopodia to mushroom spine. (C) The length of dendritic spines (a) and width of the spine head (b) in the control (GFP only) and *Auts2* KO neurons (Cre) at DIV28 were measured (control; n=155 spines, Cre; n=158 spines). (D) The measurement of PSD-95 puncta size in control (GFP only) and *Auts2* KO neurons (Cre) at DIV 21 (n=160). A.U, arbitrary unit. (E) Subcellular localization of Myc-NES- and Myc-NLS-FL-AUTS2^{R} in WT primary hippocampal neurons at DIV12. (F) WT primary hippocampal neurons were co-electroporated with GFP and expression vectors for the full-length AUTS2 (FL-AUTS2) and C-terminal AUTS2 short variants (S-AUTS2 var.1 and var.2) or control plasmid (Empty vector). The density of dendritic spines was measured at DIV24 (n = 20 dendrites). Data are mean ± SEM and box-and-whisker plots (medians with interquartile range, minimum, and maximum values are represented). *P < 0.05, ***P < 0.001, N.S, not significant. (A and C) unpaired t-test, (B and D) Mann-Whitney U test, (F) one-way ANOVA with Dunnett's post hoc test. Quantifications represent data from three independent experiments. Scale bar, 5 μm in (A, E and G) and 1 μm in (C).

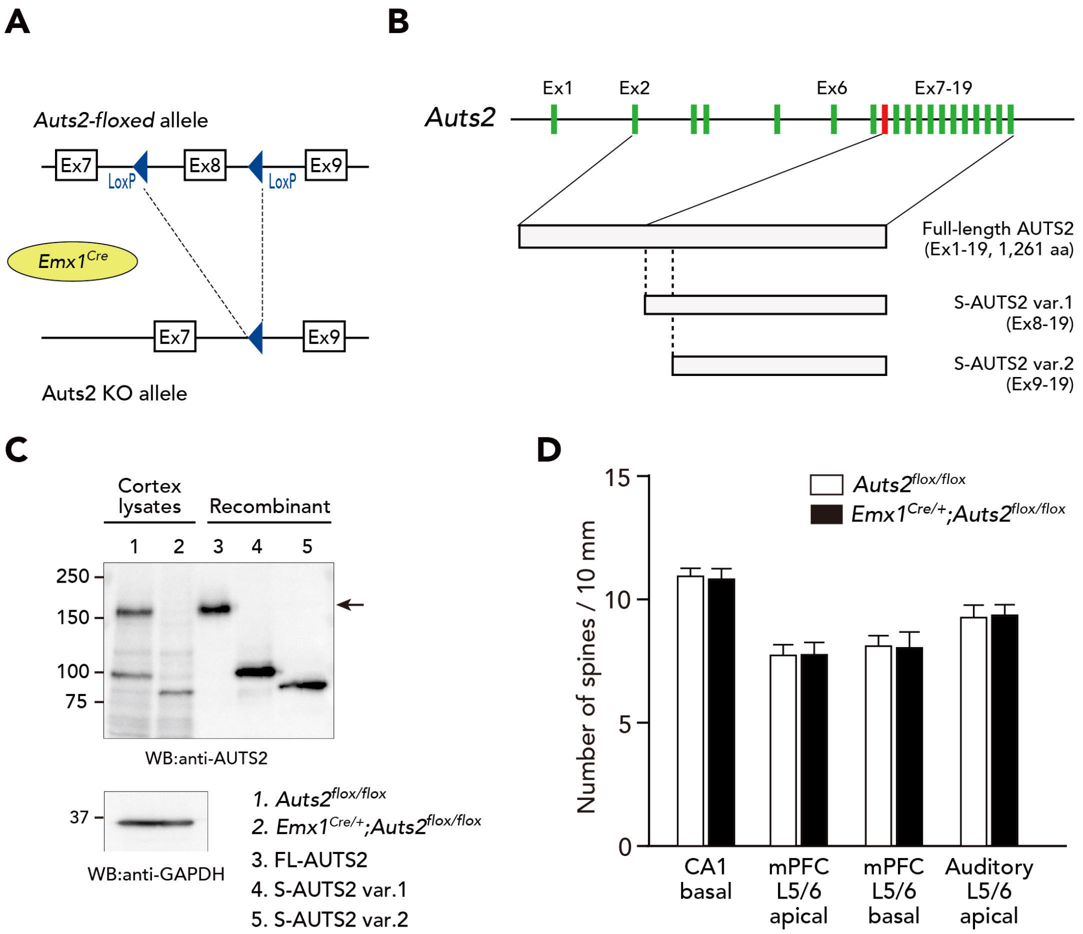

Figure S3. Analysis of spine formation in forebrain-specific *Auts2* conditional KO mice, Related to Figure 2.

(A) Schematics of the targeting strategy. The deletion of exon 8 at *Auts2* locus in pyramidal neurons of the forebrain was generated by crossing the *Auts2-floxed* mice with *Emx1*^{Cre} mice. (B) Schematic of *Auts2* genomic region and the protein structure of AUTS2 isoforms. (C) Western blotting of lysates from P0 cerebral cortex of *Auts2*$^{flox/flox}$ (Control) and *Emx1*$^{Cre/+}$;*Auts2*$^{flox/flox}$ homozygotes using anti-AUTS2 antibody. Immunoblot of lysates from HEK293T cells expressing the recombinant full-length AUTS2 and the C-terminal AUTS2 short variants (S-AUTS2 var.1 and var.2) are also shown. Full-length AUTS2 (arrow) as well as the S-AUTS2 var.1 were completely eliminated in *Auts2* homozygotic mutant cerebral cortices whereas the S-AUTS2 var.2 was alternatively increased. (D) Summary graph of the spine density on the basal dendrites of CA1 pyramidal neurons, apical and basal dendrites of the deep-layer (L5/6) neurons at mPFC and auditory cortex in the *Auts2*$^{flox/flox}$ and *Emx1*$^{Cre/+}$;*Auts2*$^{flox/flox}$ homozygotic mutant mouse brains. (n=20 dendrites from N=3 animals) Data are presented as mean ± SEM. unpaired t-test. Scale bar, 10 µm.

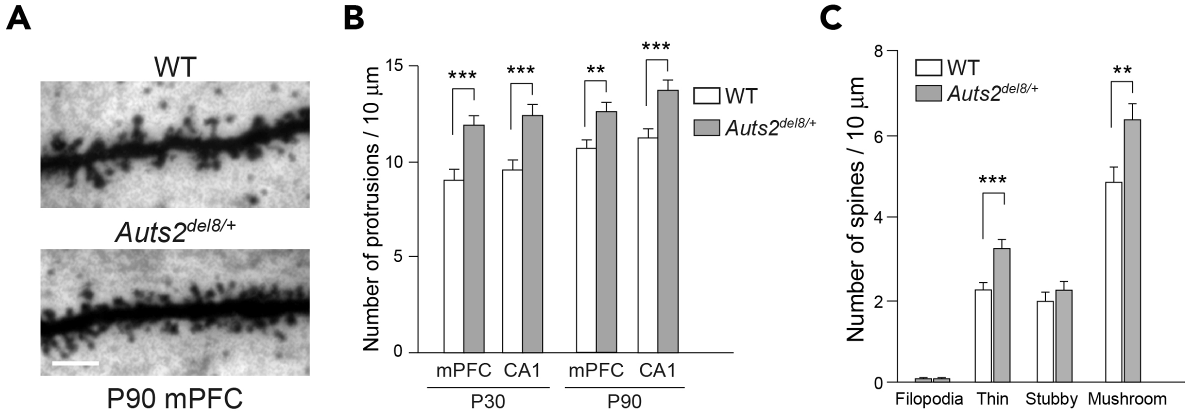

Figure S4. Spines were abnormally increased in the constitutive *Auts2*$^{del8/+}$ mutant mature brains *in vivo*, Related to Figure 2.

(A) Representative images of the dendritic spines from Golgi-stained upper-layer pyramidal neurons in the mPFC of WT (upper panel) and *Auts2*$^{del8/+}$ heterozygotic mutant mouse brains (lower panel) at P90. (B) Summary graph of the spine density on the neurons in indicated brain areas in WT and *Auts2*$^{del8/+}$ heterozygotes at young adult (P30) and mature adult (P90). (n=20 dendrites from n=3 animals). (C) The density of each category of spines in the upper-layer neurons in the mPFC was measured in WT and *Auts2*$^{del8/+}$ mutant mouse brains at P90 (n=20 dendrites from n=3 animals). Data are presented as mean ± SEM. **$P < 0.01$, **$P < 0.001$, (B) unpaired t-test, (C) Mann-Whitney U test. Scale bar, 5 µm.

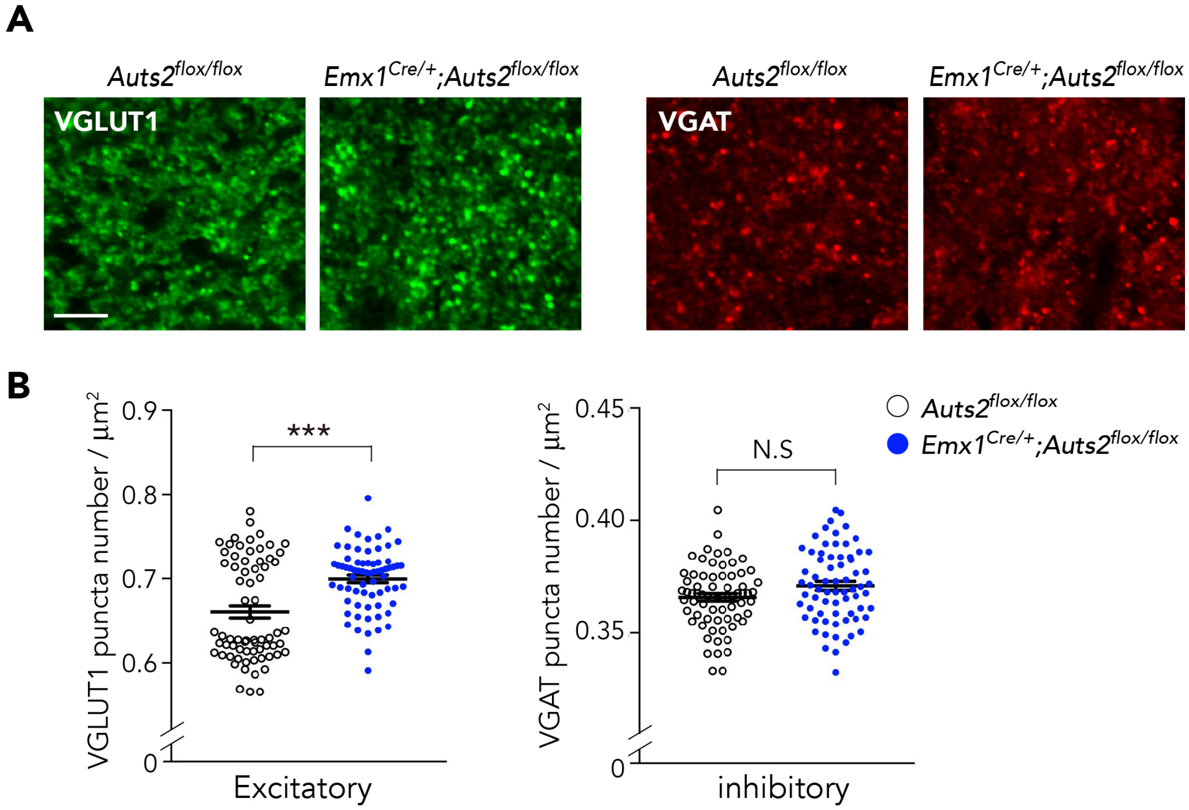

Figure S5. Loss of *Auts2* in the forebrain induces the aberrant excitatory synapse formation, Related to Figure 2.

(A) Representative images of upper-layer in medial prefrontal cortex (mPFC) sections from *Auts2* homozygotic mutant (*Emx1$^{Cre/+}$;Auts2$^{flox/flox}$*) and control (*Auts2$^{flox/flox}$*) mice at P35 stained with VGLUT1 (green) or VGAT (red), for excitatory and inhibitory presynaptic markers, respectively. (B) Quantification of the density of VGLUT1- and VGAT-positive synaptic puncta in the mPFC. Data are means ± SEM (n = 69 sections from N = 3 brains). **$P < 0.001$, N.S, not significant. (B) Mann-Whitney U test. Scale bar, 5 µm.

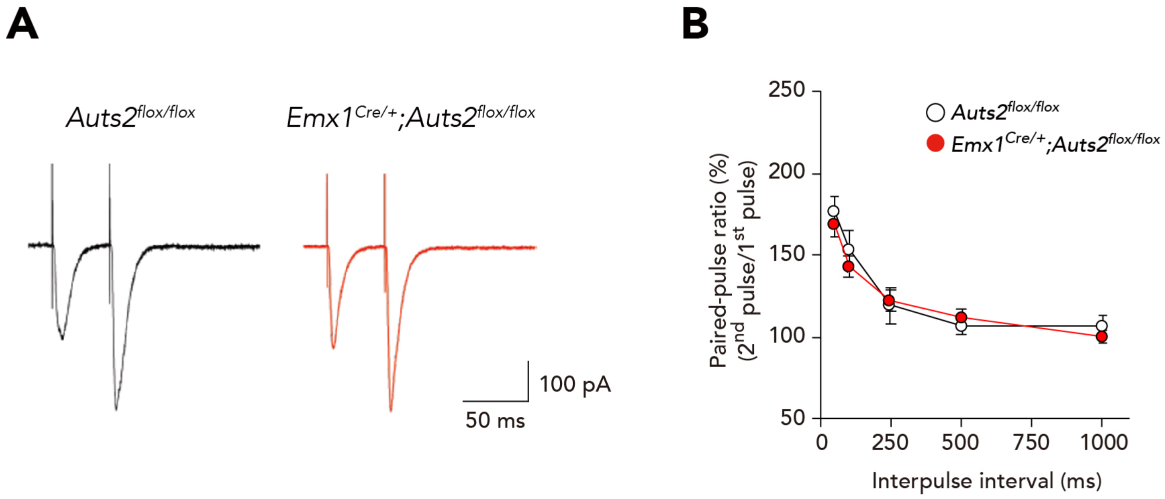

Figure 6. Electrophysiological analysis of CA1 pyramidal neurons in *Emx1^{Cre};Auts2^{flox}* mutant mice, Related to Figure 3.

(A) Representative traces showing evoked EPSCs in response to paired sets of local stimulation at Schaffer collaterals in CA1 hippocampal region. (B) Plot of the paired-pulse ratio (interpulse interval: 50 ms, 100 ms, 250 ms, 500 ms and 1000 ms), calculated as the ratio (%) of the second to first EPSC amplitude (n=12 cells from 3-4 mice). Data are mean ± SEM. (B) repeated-measure ANOVA.

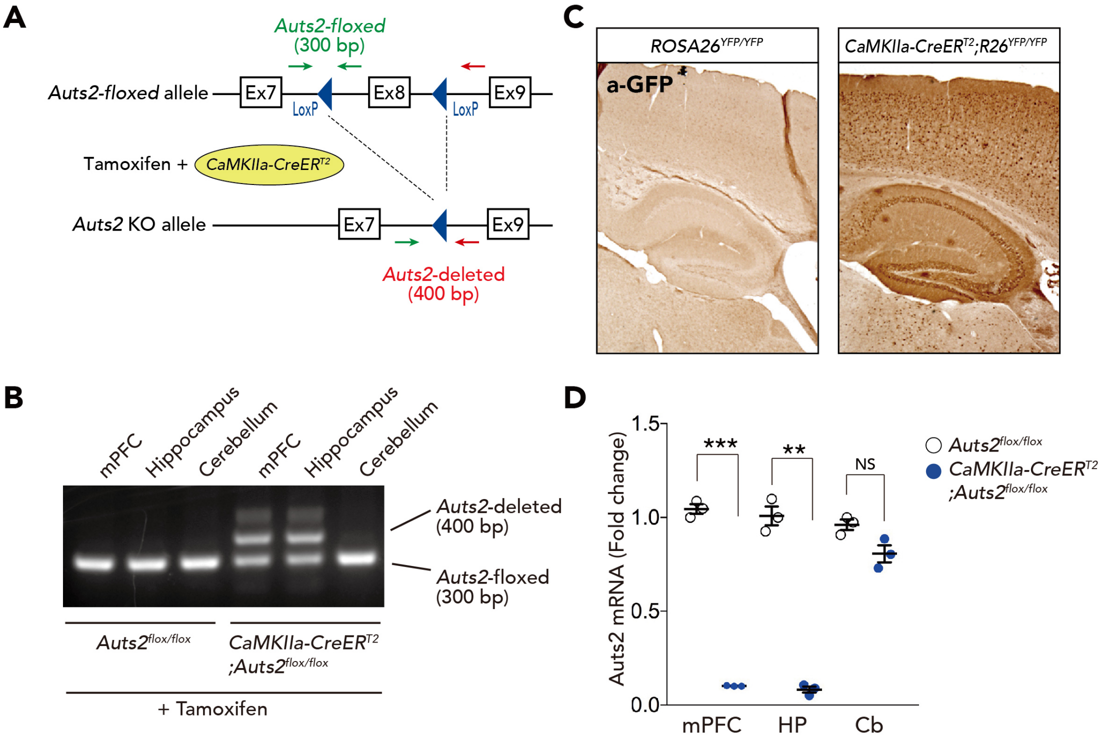

Figure S7. Conditional deletion of *Auts2* in postnatal forebrain leads to excessive spine formation, Related to Figure 4.

(A) Schematics of the targeting strategy. The inducible conditional deletion of exon 8 at *Auts2* locus in pyramidal neurons of the forebrain was generated by breeding the *Auts2*$^{flox/flox}$ mice to *CaMKIIa-CreER*^{T2} mice. (B) The forebrain-specific deletion of *Auts2* locus in the homozygous mutants (*CaMKIIa-CreER*^{T2}*;Auts2*$^{flox/flox}$) and control littermates (*Auts2*$^{flox/flox}$) after tamoxifen administration was confirmed by genomic PCR using the primer pairs indicated as green and red arrows in (A). (C) Examination of CreER^{T2} recombinase activity in the postnatal brain. Tamoxifen was administered to *CaMKIIa-CreER*^{T2}*;ROSA26R*$^{YFP/YFP}$ reporter mice and the *ROSA26R*$^{YFP/YFP}$ control littermate during P21-25. Brain sections isolated 10 days after tamoxifen treatment were DAB-stained with anti-GFP antibody. The expression of EYFP was observed in the cortex and hippocampus in the presence of the *CreER*^{T2} transgene (right panel). (D) Examination of *Auts2* transcript levels in mPFC, hippocampus (HP) and cerebellum (Cb) of adult *CaMKIIa-CreER*^{T2}*;Auts2*$^{flox/flox}$ homozygotic mutant mice and the control mice (*Auts2*$^{flox/flox}$) with tamoxifen application. qPCR was performed using primers specific for the deleted exon (n=3 brains). Data are mean ± SEM. $^{**}P < 0.01$, $^{***}P < 0.001$, unpaired t-test. Scale bar, 10 µm.

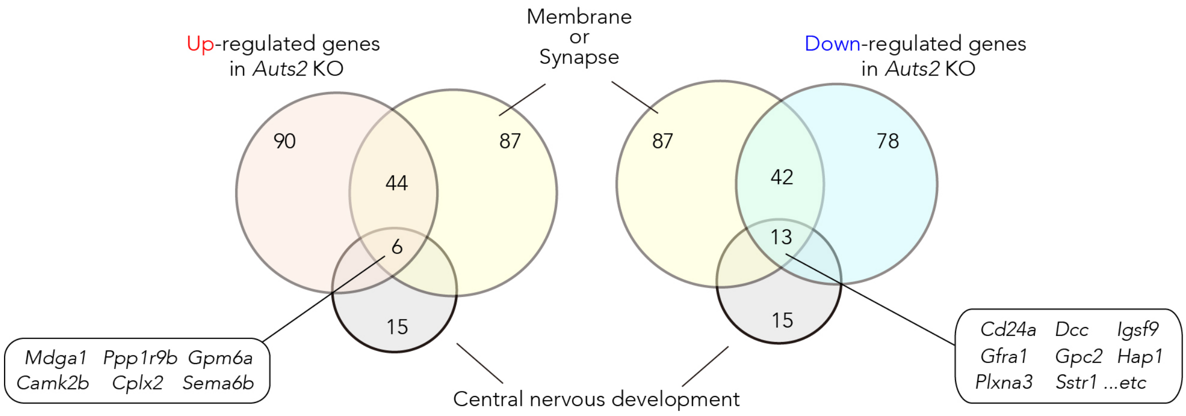

Figure S8. Transcriptome analysis of *Emx1*$^{Cre/+}$;*Auts2*$^{flox/flox}$ mutant mice hippocampal brain tissues, Related to Figure 5.

Venn diagram showing the number of up-regulated or down-regulated genes in *Auts2* KO categorized in "membrane" (GO:0016020) or "synapse" (GO:0045202) and "central nervous development" (GO:0007399).

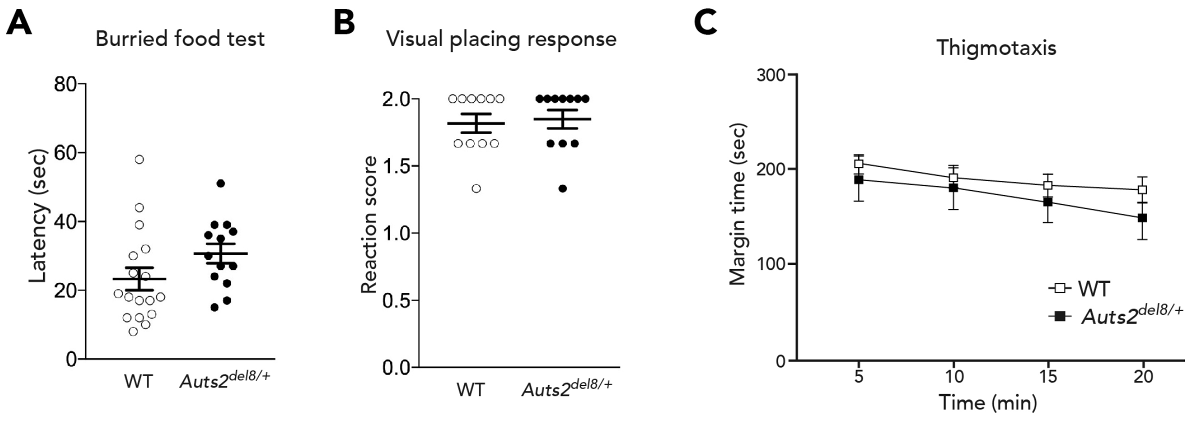

Figure S9. *Auts2*$^{del8/+}$ mutant mice exhibit normal sensory abilities, Related to Figure 6.

(A) The buried food finding test. Time spent to find buried food pellet was measured. (WT, n=17, *Auts2*$^{del8/+}$ n=13). (B) Visual placing response test. Reaction score was rated as follow: 0, no observable placing behavior; 1, a weak or delayed placing response; 2, a clear placing reaction. (WT, n=11, *Auts2*$^{del8/+}$ n=11). (C) Thigmotaxis. Time spent in the margin area of the open field box was measured every 5 min for 20 min. (WT, n=10, *Auts2*$^{del8/+}$ n=10). Data are mean ± SEM. (A and B) Mann-Whitney U test, (C) two-way ANOVA with repeated measures.

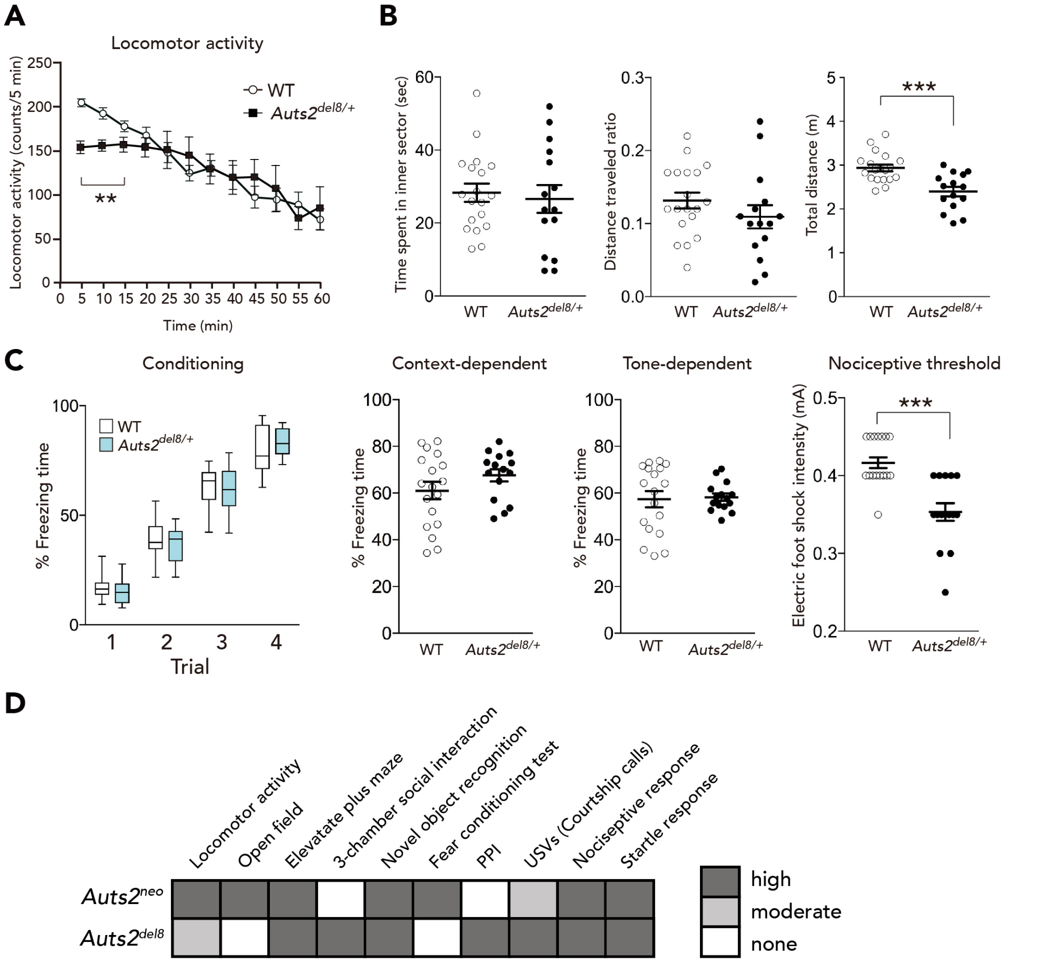

Figure S10. Behavioral analyses of $Auts2^{del8/+}$ mutant mice, Related to Figure 6.
(A) Spontaneous locomotor activity of mice in a novel environment was measured every 5 min for 60 min. $Auts2^{del8/+}$ mutant mice displayed a decrease in exploratory behavior during the first 15 min period (WT, n=19, $Auts2^{del8/+}$, n=16). (B) In open field tests, $Auts2^{del8/+}$ mutant mice exhibited a decrease in total distance traveled in a test field area for 5 min (right graph) whereas there was no significant difference between genotypes in time spent in an inner area (left graph) as well as the ratio of distance traveled in an inner area scored as the percentage of total distance traveled (middle graph) (WT, n=19, $Auts2^{del8/+}$ n=15). (C) Associative memory of WT and $Auts2^{del8/+}$ mutant mice was measured by the contextual (Context-dependent) and tone cued (Tone-dependent) fear-conditioning test 24 hrs after the conditioning phase (Conditioning). Freezing responses of $Auts2^{del8/+}$ mice during contextual and cued memory test were comparable to WT mice while *Auts2* mutant mice exhibited a higher response to lower nociceptive stimuli relative to WT mice (Nociceptive threshold) (WT, n=18, $Auts2^{del8/+}$, n=15). (D) Summary of the results from behavioral test battery for $Auts2^{neo/+}$ (Hori et al., 2015) and $Auts2^{del8/+}$ mutant mice. Data are mean ± SEM and box-and-whisker plots (medians with interquartile range, minimum, and maximum values are represented). $^{**}P < 0.01$, $^{***}P < 0.001$, (A) two-way ANOVA with repeated measures, (B) unpaired t-test, (C) two-way ANOVA with repeated measures in conditioning and Mann-Whitney U test in freezing responses.

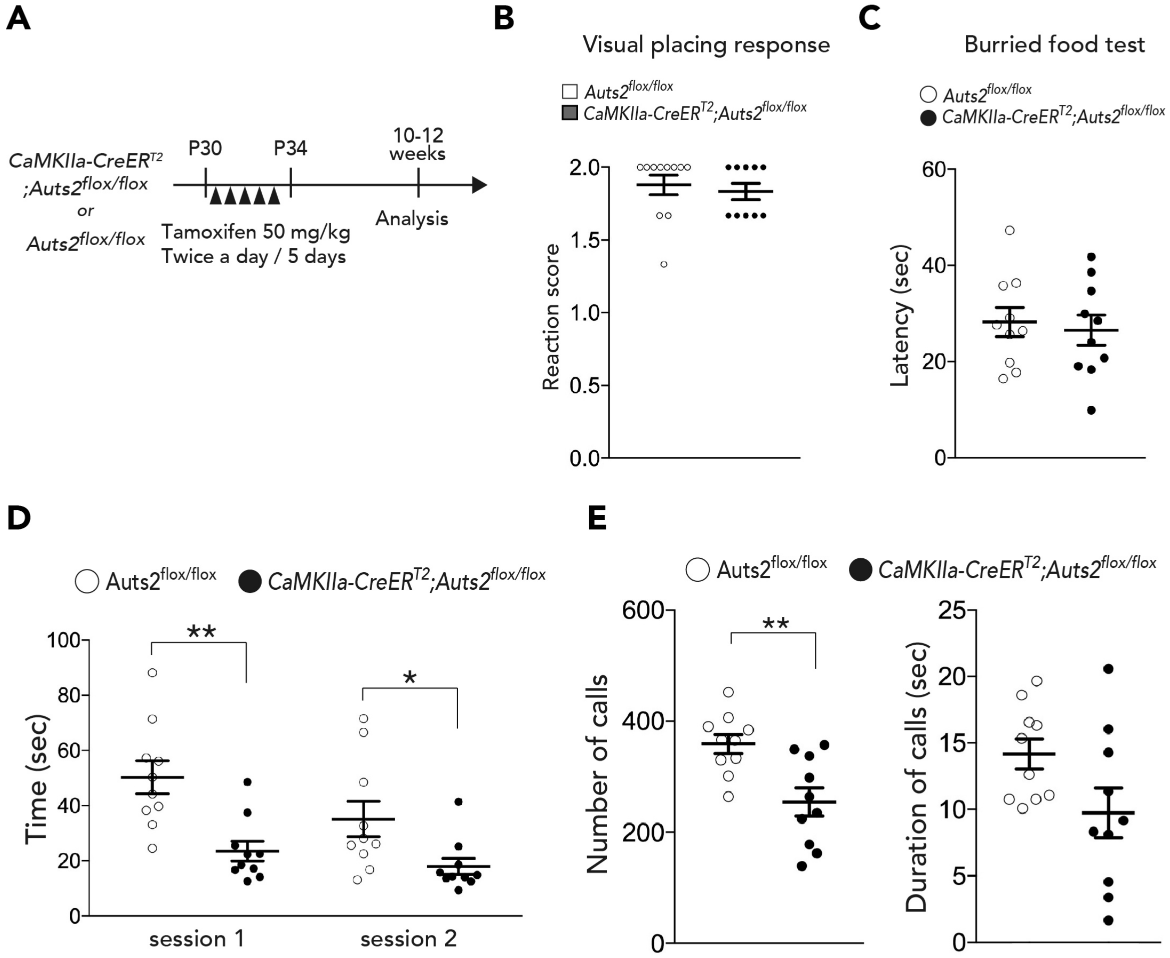

Figure S11. Behavioral analyses of *CaMKIIa-CreER^{T2};Auts2$^{flox/flox}$* mice, Related to Figure 6 and 7.

(A) Scheme illustrating the tamoxifen-inducible deletion of *Auts2* in postnatal forebrain. Tamoxifen was administered to *CaMKIIa-CreER^{T2};Auts2$^{flox/flox}$* homozygotes and their control *Auts2$^{flox/flox}$* littermate mice during P30-34 and behavioral analyses were performed during 10-12 weeks. (B) Visual placing response test. Reaction score was rated as follow: 0, no observable placing behavior; 1, a weak or delayed placing response; 2, a clear placing reaction. (*Auts2$^{flox/flox}$*, n=11, *CaMKIIa-CreER^{T2};Auts2$^{flox/flox}$* n=10). (C) The buried food finding test. Time spent to find buried food pellet was measured. (*Auts2$^{flox/flox}$*, n=10, *CaMKIIa-CreER^{T2};Auts2$^{flox/flox}$*, n=10). (D) Reciprocal social interaction test. Social interaction between *Auts2$^{flox/flox}$* or *CaMKIIa-CreER^{T2};Auts2$^{flox/flox}$* mouse pairs during 5 min were measured (*Auts2$^{flox/flox}$*, n=10, *CaMKIIa-CreER^{T2};Auts2$^{flox/flox}$* n=10). (E) The number (left) and duration (right) of USVs during 1 min in adult *Auts2$^{flox/flox}$* or *CaMKIIa-CreER^{T2};Auts2$^{flox/flox}$* mice (*Auts2$^{flox/flox}$*, n=10, *CaMKIIa-CreER^{T2};Auts2$^{flox/flox}$* n=10). Data are the means ± SEM. *$P < 0.05$, **$P < 0.01$, (B ,C and E) unpaired t-test, (D) two-way ANOVA.

Mouse strain	Allele type	Genotype
Auts2^{neo}	Global KO<br>(*Neo* gene knock-in)	WT (Control)<br>*Auts2*$^{Neo/+}$ (Heterozygote)
Auts2^{del8}	Global KO<br>(Exon 8 deleted)	WT (Control)<br>*Auts2*$^{del8/+}$ (Heterozygote)
Emx1^{Cre};*Auts2*^{flox}	forebrain-specific<br>conditional KO<br>(Exon 8 deleted)	*Auts2*$^{flox/flox}$ (Control)<br>*Emx1*$^{Cre/+}$;*Auts2*$^{flox/+}$ (Heterozygote)<br>*Emx1*$^{Cre/+}$;*Auts2*$^{flox/flox}$ (Homozygote)
CaMKIIa-CreER^{T2};*Auts2*^{flox}	Tamoxifen-inducible<br>mature projection neuron<br>-specific conditional KO<br>(Exon 8 deleted)	*Auts2*$^{flox/flox}$ (Control)<br>*CaMKIIa-CreER*^{T2};*Auts2*$^{flox/flox}$<br>(Homozygote)

Table S1. Summary of *Auts2* mutant mouse strains, Related to Figure 2, 4, 6 and 7.

Transparent Methods

Experimental animals

Rosa26R^{YFP} mouse line (stock no. 006148) was obtained from The Jackson Laboratory. Genotyping for the *Auts2-floxed* mice with a pure C57BL/6N genetic background was performed by PCR with the following primers: (F) 5′-GGCAGGTGGTTAGGTTACA-3′; (R) 5′-CAGTGCTAGAATCACAGCTG-3′. For *Auts2*^{del8} mice with C57BL/6N background, the following genotyping primer was used with the above two primers: 5′-ATCTTGGGTTCCTTCCTCAG-3′ (Table S1) (Hori et al., 2014). *Emx1*^{Cre} (stock #RBRC00808, C57BL/6J background) and *CaMKIIa-CreER*^{T2} (B6.FVB-Tg(Camk2a-cre/ERT2)2Gsc/Ieg, stock #EM02125) mice were purchased from RIKEN BioResource Center (RIKEN, Tsukuba, Japan) and European Mouse Mutant Archive (EMMA) (HelmholtzZentrum München, Neuherberg, Germany), respectively (Erdmann et al., 2007; Iwasato et al., 2000). *Emx1*^{Cre} mice were backcrossed for 8 generations to C57BL6/N wild type mice (Charles River Laboratories, Kanagawa, Japan) before crossing with *Auts2*$^{flox/flox}$ mice. *Emx1*$^{Cre/+}$;*Auts2*$^{flox/flox}$ homozygous mutant mice were generated by crossing *Emx1*$^{Cre/+}$ mice with *Auts2*$^{flox/flox}$ mice to yield *Emx1*$^{Cre/+}$:*Auts2*$^{flox/+}$ heterozygous mutant progeny. *Emx1*$^{Cre/+}$;*Auts2*$^{flox/+}$ male mice were then crossed with *Auts2*$^{flox/flox}$ female mice to obtain litters consisting of control (*Auts2*$^{flox/+}$or *Auts2*$^{flox/flox}$) mice, heterozygous (*Emx1*$^{Cre/+}$;*Auts2*$^{flox/+}$) or homozygous (*Emx1*$^{Cre/+}$;*Auts2*$^{flox/flox}$) mutant mice. In this study, *Auts2*$^{flox/flox}$ mice were used as the controls. For the generation of *CaMKIIa-CreER*^{T2};*Auts2*$^{flox/flox}$ mice, *CaMKIIa-CreER*^{T2} mice with mixed genetic background (F1: C57BL6N/FVB) were first crossed with *Auts2*$^{flox/flox}$ mice (C57BL/6N background) to obtain *CaMKIIa-CreER*^{T2};*Auts2*$^{flox/+}$ mice. *CaMKIIa-CreER*^{T2};*Auts2*$^{flox/+}$ mice were then crossed with *Auts2*$^{flox/flox}$ mice to obtain litters consisting of *Auts2*$^{flox/+}$, *Auts2*$^{flox/flox}$, *CaMKIIa-CreER*^{T2};*Auts2*$^{flox/+}$ and *CaMKIIa-CreER*^{T2};*Auts2*$^{flox/flox}$ mice. For all experiments, the *CaMKIIa-CreER*^{T2};*Auts2*$^{flox/flox}$ males were crossed with *Auts2*$^{flox/flox}$ females to yield the test animal cohorts consisting of the *CaMKIIa-CreER*^{T2};*Auts2*$^{flox/flox}$ and *Auts2*$^{flox/flox}$ littermates. All tested animals in the behavioral analyses were generated through at least 9 crosses with C57BL/6N background animals (e.g. *Auts2*$^{flox/flox}$) to obtain genetic backgrounds close to that of C57BL/6N. For the experiments with *CaMKIIa-CreER*^{T2} mice, tamoxifen (Sigma-Aldrich, St. Louis, MO, USA) was administered at 50 mg/kg during postnatal 21-25 days for anatomical analysis or P30-34 for behavioral analyses by intraperitoneal injection twice daily for 5 consecutive days and the analyses were performed at postnatal day 50 and 10-12 weeks, respectively. Mice were maintained in ventilated racks under a 12-h light/dark cycle with food and water ad libitum in temperature controlled, pathogen-free facilities. Mice of each genotype were randomly allocated to different experiments. Both male and female animals were used in anatomical and electrophysiological experiments. All animal experiments in this study have been approved by the Animal Care and Use Committee of the National Institute of Neuroscience, Japan, and the

guidelines established by the Institutional Animal Care and Use Committee of Nagoya University, the Guiding Principles for the Care and Use of Laboratory Animals approved by the Japanese Pharmacological Society, and the National Institutes of Health Guide for the Care and Use of Laboratory Animals. All efforts were made to minimize suffering and to reduce the number of animals used.

Behavioral analysis

For behavioral test battery using *Auts2*^{del8} mice, two independent cohorts of *Auts2*$^{del8/+}$ heterozygotes and their wild type littermate male mice (8-19 weeks old) were tested, to confirm findings. All behavioral tests using *Auts2*^{del8} mice were obtained by crossing *Auts2*$^{del8/+}$ heterozygous male mice with wild type C57BL6/N female mice (Charles River Laboratories, Kanagawa, Japan) to avoid the possibility that altered behaviors in the mutant dams could influence the postnatal development of their offspring. After weaning, male mice were cohoused in same-genotype groups of 2-4 littermates per cage before and during the behavioral tests.

Behavioral tests were performed using the same set of mice in the following sequence: locomotor activity, open field test, novel object recognition test, elevated plus maze, 3-chamber social interaction test, prepulse inhibition test, and fear conditioning test. For recording of USVs, buried food finding test, visual placing response test, thigmotaxis and reciprocal social interaction test, separate cohorts of mice were used.

For behavioral analyses using *CaMKIIa-CreER*^{T2}*;Auts2*^{flox} conditional KO mice, *CaMKIIa-CreER*^{T2}*;Auts2*$^{flox/flox}$ homozygous mutant mice and their control littermate *Auts2*$^{flox/flox}$ male mice were used.

The buried food finding test

The buried food finding test was carried out as described below (Yang and Crawley, 2009). Male mice were fasted for 18-24 hrs before testing. Subject mice were individually habituated in a clean cage (45 x 23 x 15 cm) for 5 min. For testing, a food pellet was buried at the end of the cage under 1 cm of wood-chip bedding. Subject mice were placed in the corner opposite to the site of the concealed food pellet. Movement of mice was recorded by video camera and time spent to explore the food pellet was measured by an examiner with stopwatch.

Visual placing response test

The function of the visual system was evaluated by the visual placing response according to the methods by Metz and Schwab (Metz and Schwab, 2004). In this test, the test mouse was suspended by its tail and lowered toward a solid object without any contact to the vibrissae. When the head of a mouse approaches near the edge of the object, the mouse normally raises its head and extends the forelimbs to place them onto the object. The procedure was conducted by three trials and the mean response was rated with the following scoring system: 0 indicates no observable placing behavior, 1 represents a weak or delayed placing response and 2 points indicates a clear placing reaction.

Whisker twitch reflex

The whisker twitch reflex was tested by approaching from behind and lightly touching one set of vibrissae, eliciting head turning to the side on which the vibrissae was touched (Miyakawa et al., 2001).

Thigmotaxis

Mice were placed in the center of the test chamber (26 cm x 26 cm x 40 cm) under moderately bright light conditions (100 lux) and allowed to explore it. Each 20 min session was monitored by video camera and analyzed in four 5 min bins. Time spent in the marginal area defined as a 4 cm band extending from the wall was measured by examiner with a stopwatch.

Locomotor activity

Spontaneous exploratory locomotion was examined as fallows (Nagai et al., 2010). Mice were individually placed in a transparent acrylic cage with a black frosted Plexiglas floor (25×25×20 cm) under moderate light conditions (15 lux), and locomotor activity was measured every 5 min for 60 min using digital counters with an infrared sensor (BrainScience Idea, Osaka, Japan).

Open field test

Mice were placed in the center of the test chamber (diameter, 60 cm; height, 35 cm) under moderate light conditions (60 lux) and allowed to explore it for 5 min, while their activity was automatically analyzed using the ethovision automated tracking program (Brainscience Idea Co.

Ltd., Osaka, Japan) (Lee et al., 2005). The center zone of the open-field was defined as the 40 cm-diameter inner circle in the chamber. Movements were measured via a camera mounted above the open field. Measurements included distance traveled and time spent in the inner and outer sections.

Elevated-plus maze test

The apparatus consists of two open (25 × 8 × 0.5 cm), two closed (25 × 8 × 20 cm) arms and a common central zone (8 × 8 cm) to form a plus shape (Koike et al., 2009). The entire apparatus was elevated to 50 cm above floor level under moderately bright conditions (170 lux). The test begins by placing a mouse in the central zone of the maze facing an open arm. The animals were allowed to move freely in the maze for 5 min. An arm entry was defined as all four paws in the arm. The duration of time spent in an arm and number of arm entries is measured. Animal falling off open arms was excluded from analyses.

Reciprocal social interaction test

Reciprocal social interaction test was performed as described below (Harper et al., 2012; Hiramoto et al., 2011). Age-matched, non-littermate male WT and *Auts2*$^{del8/+}$ mutant mouse pairs, or *CaMKIIa-CreER*^{T2}*;Auts2*$^{flox/flox}$ and *Auts2*$^{flox/flox}$ control mice pairs were tested. Each individual mouse was placed in a new cage in the experimental room for 30 min. Pairs of unfamiliar mice (non-cage mate and non-littermate) with age-matched, same genotype were then placed in a new, third cage (45 x 23 x 15 cm) for two 5 min sessions with a 30 min interval. Behaviors were monitored with video camera and time spent in active behaviors were analyzed by examiner with a stopwatch. Active social behaviors included aggressive forms (i.e. wrestling, boxing, kicks, mounting, tail rattle, bites, sideway offense and pursuit) and affiliative forms (i.e. olfactory investigation and allogrooming).

Three-chamber social interaction test

A three-chamber arena was used to examine social approach and preference for social novelty as follows (Nadler et al., 2004). During habituation, empty cylinders were placed in each end chamber. The test subject was placed in the center chamber and its behavioral approach to the chambers was monitored for 10 min. During the sociability test, an unfamiliar male C57BL/6N

mouse (stranger 1) that had no prior contact with the test mouse was put in one of the empty chambers, and the behavioral approach to the empty chamber and stranger 1 was monitored for 10 min. During the social novelty test, new unfamiliar male C57BL/6N mouse (stranger 2) was placed in another chamber, and the behavioral approach to the stranger 1 and stranger 2 was monitored for additional 10 min. The amount of time spent in each arena was measured by an ethovision automated tracking program (Noldus, Wageningen, Netherlands).

Novel object recognition test

A novel object recognition test was carried out as described below (Nagai et al., 2007). Mice were individually habituated to an open box (30 × 30 × 35 cm) for 3 days. All sessions were performed under conditions of illumination (16 lux). During the training session, two novel objects of similar size were placed in the open box and mice were allowed to explore for 10 min. The objects were a golf ball, wooden cylinder, and square pyramid, which were different in shape and color. An animal was considered to be exploring the object when its head was facing the object or it was touching or sniffing the object. The time spent exploring each object was recorded by using video camera and analyzed in a double-blind manner. During retention sessions, mice were placed back into the same box 24 h after the training session, one of the familiar objects used during training session was replaced by a novel object, and the mice were allowed to explore the two objects freely for 5 min. The exploratory index in the retention session, the ratio of the amount of time spent exploring the novel object to the total time spent exploring both objects, was used to measure cognitive function. In the training session, the preference index was calculated as the ratio of time spent exploring the object that was replaced by a novel object in the retention session to the total exploration time.

Cued and contextual fear conditioning test

Cued and contextual fear conditioning test was carried out as described below (Ibi et al., 2010). Training took place in the chamber (30 × 30 × 40 cm) equipped with a metal floor and a 15-sec white noise tone (85 dB) was delivered (conditioned stimulus). During the last 5 sec of the tone stimulus, a foot shock of 0.8 mA was delivered through a shock generator as an unconditioned stimulus (Brainscience Idea Co. Ltd., Osaka, Japan). This procedure was repeated four times at 15-sec intervals. Twenty-four hr after conditioning, the context-dependent test was

performed. For the context-dependent test, each mouse was put in the training chamber, and the freezing response was monitored for 2 min in the absence of the conditioned stimulus. Tone-dependent testing was performed 4 hr after the context-dependent test. For the tone-dependent test, the freezing response was measured for 1 min in a standard transparent rectangular rodent cage (25 × 30 × 18 cm) in the presence of a continuous-tone stimulus identical to the conditioned stimulus using mice that had been subjected to the context-dependent test.

Prepulse inhibition (PPI) test

The PPI test was carried out as follows (Takahashi et al., 2007). The animals were placed in the chamber (San Diego Instruments, San Diego, California) and were habituated for 10 min. During the habituation time, 65 dB background white noise was delivered. Mice then received 10 startle trials, 10 no-stimulus trials and 40 PPI trials. The intertrial stimulus intervals were between 10 and 20 sec and the total session lasted 17 min. Mice were presented with a single 120 dB white noise burst lasting 40 msec during the startle trial. PPI trials consisted of a prepulse (20 msec burst of white noise at 69, 73, 77 or 81 dB intensity) followed, 100 msec later, by the startle stimulus (120 dB, 40 msec white noise). Each of the four prepulse trials (69, 73, 77 or 81 dB) was performed 10 times. Sixty different trials were pseudo-randomly delivered, ensuring that each trial was carried out 10 times and that no two consecutive trials were identical. The resulting movement of the animal in the startle chamber was measured for 100 msec after startle stimulus onset (sampling frequency 1 kHz), rectified, amplified and fed into a computer, which calculated the maximal response over the 100 msec. Basal startle amplitude was determined as the mean amplitude of the 10 startle trials. PPI was calculated according to the following formula: 100 × [1 − (PPx/P120)] %, in which PPx is the mean amplitude of the 10 PPI trials (PP69, PP73, PP75 or PP80) and P120 is the basal startle amplitude.

Ultrasonic vocalizations

Ultrasonic Vocalizations were recorded using an UltraSoundGate system (Avisoft bioacoustics, Glienicke, Germany) composed of a CM16/CMPA condenser microphone, Avisoft-UltraSoundGate 116H computer interface, and Avisoft Recorder software with a sampling rate of 400 kHz. A microphone was hung 16 cm above the floor of a sound attenuating chamber. For

the test, male mice were individually housed in Plexiglas cages (23 cm x 16 cm x 12 cm) for a week prior to test time to acclimate to the testing environment. Unfamiliar wild type three month old C57BL6/N female mice were placed into the test male cage and recordings begun after USV was detected and continued for 1 min period.

Syllable analysis

Vocal signals recorded in wav files were automatically detected by MATLAB-based software USVSEG with modification to mouse USVs (Tachibana et al., 2014). This software segments each syllable and exports as individual jpeg files. As Tachibana et al reported (Tachibana et al., 2020), USVSEG can detect correct vocal signals with approximately 95% accuracies compared to the information that was manually defined by a human examiner. The number of USVs and duration of each call are automatically detected. By observing jpeg files, experimenters then manually excluded the files of vocalizations that includes only click-like sounds without any tone-like signals or that could not be classified into any of the call types as noises (false positive).

The vocalizations were manually categorized into 12 types observing these jpeg files based on the previously published criteria with some modification (Kikusui et al., 2011; Yasumura et al., 2014). In the previous criteria, syllables including both jumps and harmonics were classified into One Jump or More jump. In our present methods, such syllables were classified in more detailed manner in order to demonstrate more clearly whether such Jumps include harmonics or not. In addition, these 12 call types were grouped into "simple" and "complicated" syllable types based on call duration, frequency modulation and the presence/absence of harmonics or jumps. The call classifications we used are as follows;

Upward. Syllables with upwardly modulated frequency change (> 5 kHz)

Downward. Syllables with downwardly modulated frequency change (> 5 kHz).

Flat. Syllables with few frequency modification ≤ 5 kHz.

Short. syllables which is shorter than or equal to 5 msec.

Chevron. Syllables with an upsweep (greater than 5 kHz) followed by a down-sweep (greater than half of the frequency change of the upsweep) or reversed one, formed like a U or a reversed U.

Wave. Syllables with two directional changes in frequency (> 5 kHz).

Complex. Syllables with three or more directional changes in frequency (> 5 kHz).

One jump. Syllables with one frequency jump and no time gap before and after jump.
More jump. Syllables with two or more frequency jumps and no time gap before and after jumps.
Harmonics. syllables that were displayed as one main component stacking with other harmonically components of different frequency (without jumps).
One jump + harmonics. Syllables with components of both One jump and Harmonics.
More jump + harmonics. Syllables with components of both More jump and Harmonics.

Plasmid construction

The plasmid construction of pCAG-Myc-AUTS2-full length, FL-AUTS2^{R} (the shRNA-resistant AUTS2-full length), NES-FL-AUTS2^{R} and S-AUTS2-var.2 were described previously (Hori et al., 2014). cDNA fragments for S-AUTS2-var.1 encoding 1,372-3,786 bp were amplified by PCR using full-length *Auts2* cDNA as a template and subcloned into pCAGGS vector. To construct the 3xNLS-AUTS2 expression plasmid, two oligonucleotides coding the three tandem nuclear localization signal (NLS) sequence of the SV40 Large T-antigen (PKKKRKV) were annealed and inserted between Myc-tag and 5′-terminus of AUTS2 ORF in pCAG-Myc-AUTS2^{R} with EcoRI site: Fwd, (5′-AATTGGTGCACGTGGATCCAAAAAAGAAGAGAAAGGTAGATCCAAAAAAGAAGAGAAAGGTAGATCCAAAAAAGAAGAGAAAGGTACACGTGTCCG-3′): Rev, (5′-AATTCGGACACGTGTACCTTTCTCTTCTTTTTTGGATCTACCTTTCTCTTCTTTTTTGGATCTACCTTTCTCTTCTTTTTTGGATCCACGTGCACC-3′). The expression plasmids for EGFP, Cre recombinase and shRNAs were described previously (Hori et al., 2014).

Primary culture of hippocampal neurons

Primary hippocampal cultures were prepared as described below (Hori et al., 2014; Hori et al., 2005). Hippocampi at E17.5 were dissected from C57BL6/N wild type or homozygotic *Auts*$^{2flox/flox}$ mouse embryos and dissociated using Neuron Dissociation Solution S (Fujifilm Wako Pure Chemical Corporation, Osaka, Japan). The dispersed neurons were electroporated with the expression plasmids or shRNA vectors using the NEPA21 electroporator (Nepa Gene, Chiba, Japan) according to the manufacturer’s instructions. The electroporated neurons were mixed with the transfection-free control neurons at a ratio of approximately 1:20 and plated on coverslips coated with 0.1 mg/ml poly-D-lysine (Sigma-Aldrich, St. Louis, MO, USA) at a density

of 8,000 – 12,000 cells/cm^2 and maintained in astroglial-conditioned Neurobasal medium containing 2% B27 supplement (Thermo Fisher Scientific, Waltham, MA, USA) and 1 mM L-glutamine.

Immunostaining

For immunocytochemistry, cells were fixed with 4% PFA / 4% sucrose for 40 min on ice. Immunostaining was performed using the following primary antibodies: mouse anti-PSD-95 (6G6-1C9, ThermoFisher Scientific, Waltham, MA, USA), mouse anti-Gephyrin (3B11, Synaptic Systems, Goettingen, Germany), rabbit anti-Synapsin-1 (D12G5, Cell Signaling Technology, MA, USA), rat anti-GFP (RQ1, gift from A. Imura, BRI, Kobe), mouse anti-Myc-tag (M192-3, MBL, Nagoya, Japan). For immunohistochemistry, adult mouse brains were dissected after mice were deeply anesthetized and transcardially perfused with 4% PFA. The brains were post-fixed with 4% PFA / 5% sucrose for 6 hrs or overnight at 4 °C, rinsed with PBS, cryoprotected with 30% sucrose, embedded in O.C.T compound (Sakura Fine-Tek, Tokyo, Japan), and cryosectioned at 14~30 μm. For presynaptic marker staining, tissue sections were blocked with 5% normal donkey serum / 1% BSA and 0.1% Triton X100 in PBS and immunolabeled with guinea pig anti-VGLUT1 (AB5905, Merck Millipore, Burlington, MA, USA) and rabbit anti-VGAT (AB5062P, Merck Millipore, Burlington, MA, USA) antibodies overnight at 4 °C. For quantification of presynaptic puncta number, fluorescence images of mPFC regions were acquired using 100x objective equipped with a confocal laser scanning microscope FV1000 (Olympus, Tokyo, Japan), magnified three fold and analyzed using the "analyze particle" module in Fiji-imageJ software (Schindelin et al., 2012).

For c-Fos staining, tissue sections were immunostained with rabbit anti-c-Fos antibody (sc-52, Santa Cruz Biotechnology, Inc., Dallas, TX, USA). Acquisition of fluorescent images, counts and measurement of dendritic protrusions were carried out using a Zeiss LSM 780 confocal microscope system and ZEN software (Carl Zeiss, Oberkochen, Germany). For analysis of dendritic arbors, images were acquired using a Plan-Apochromat 20×/0.8 differential interference contrast (DIC) objective. Tracing, measurement of dendritic length and Sholl analysis were performed using Neurolucida software (MBF Bioscience, Williston, VT, USA). For analysis of dendritic spines, compiled z-stack images were acquired using a Plan-Apochromat 63×/1.40 oil-immersion DIC objective at 0.37 μm intervals, sampling above and below the

dendrites. Maximum intensity projections were used for quantification. For DAB staining with rat anti-GFP antibody (RQ1), the sections were processed using the VECTASTAIN ABC system (Vector Laboratories, Burlingame, CA, USA) with diaminobenzidine (Sigma-Aldrich, St. Louis, MO, USA) and images were taken on Keyence All-in-One fluorescence microscope (BZ-X700, Osaka, Japan).

Immunoblotting

The lysates of HEK293T cells transfected with AUTS2 expression plasmids or cerebral cortices from mouse brain at P0 were solubilized in SDS sample buffer and separated in 2-15% gradient gel by SDS-PAGE (Gellex International co.ltd., Tokyo, Japan). Proteins transferred onto a PVDF membrane were immunoblotted with anti-AUTS2 antibody (HPA000390, Sigma-Aldrich, St. Louis, MO, USA) and anti-GAPDH (2118S, Cell Signaling Technology, Tokyo, Japan) antibodies, and visualized using HRP-conjugated secondary antibody (GE Healthcare, Chicago, IL, USA) followed by ECL Prime (GE Healthcare, Chicago, IL, USA). Signals were detected with a cooled CCD camera (LAS-4000 mini; Fujifilm, Kanagawa, Japan).

Golgi-staining

Whole brains collected from mice were subjected to Golgi impregnation solution (FD Rapid GolgiStain kit, FD NeuroTechnologies, Columbia MD, USA). Coronal sections with 80-100 μm thick were obtained with cryostat and mounted on gelatin-coated slides. After tissues were processed for Golgi-Cox staining according to manufacturer's instructions, the brain sections were dehydrated with a graded series of ethanol, immersed in xylene, and embedded in Entellan (Merck, Darmstadt, Germany). Neurons were traced under bright-field using a Leica microscope (DM5000B, Leica Microsystems, Danaher, Germany) with a 100x oil-immersion objective and were 3D-reconstructed by Neurolucida Software.

Spine measurements

For primary cultured neurons, spines or synaptic puncta immuno-labeled with synapse markers along secondary dendritic segments randomly selected (>30 μm length) were counted. For Golgi-stained tissue samples, spines along primary apical dendrites immediately proximal to the cell soma (0 – 50 μm) of cortical layer II/III and CA1 hippocampal pyramidal neurons, or

spines along secondary dendrites within 100 μm from the soma of the neurons indicated in the figure were examined. Spine densities were calculated as mean number of spines per 10 μm dendrites. On the basis of spine morphology, dendritic protrusions were classified into the following four categories (Harris and Kater, 1994): thin (≥ 0.5 μm protrusions with small bulbous head less than twice as large as spine neck), mushroom (≥ 0.5 μm protrusions with bulbous head more than twice as large as spine neck), stubby (≥ 0.5 μm protrusions with bulbous head but without a neck), filopodia (≥ 5 μm long and thin protrusions without bulbous heads). Dendritic protrusions with total lengths exceeding 10 μm were considered as branched dendrites and excluded from the analysis. Z-stack images of dendritic spines were taken using a Keyence All-in-One microscope with a 40x objective (BZ-X700, Osaka, Japan).

Electrophysiology

Whole-cell voltage-clamp recordings of mEPSCs and mIPSCs using brain slices were conducted as follows(Takahashi et al., 2012). Coronal hippocampal slices with 400 μm thickness from adult mice at P33-44 were prepared in ice-cold dissection buffer (300 mM sucrose, 3.4 mM KCl, 0.6 mM NaH_2PO_4, 10 mM D-Glucose, 10 mM HEPES, 3.0 mM $MgCl_2$, 0.3 mM $CaCl_2$ at pH 7.4) using a VT1200S vibratome (Leica Biosystems, Danaher, Germany). Hippocampal slices were incubated in artificial cerebrospinal fluid (ACSF; 119 mM NaCl, 2.5 mM KCl, 1.0 mM NaH_2PO_4, 26.2 mM $NaHCO_3$, 11 mM D-Glucose, 4.0 mM $MgSO_4$, 4.0 mM $CaCl_2$, gassed with 95% O_2 and 5% CO_2), left to recover for more than 1 hour at room temperature, and then transferred to a recording chamber mounted on an upright microscope (BX61WI, Olympus, Tokyo, Japan). For voltage-clamp recordings of hippocampal slices, borosilicate glass pipettes (4-6 MΩ) were filled with the internal solutions (135 mM $CsMeSO_4$, 10 mM HEPES, 0.2 mM EGTA, 8 mM NaCl, 4 mM Mg-ATP, 0.3 mM Na_3GTP at pH7.2, osmolality adjusted to 280-300 mOsm). All data of whole-cell voltage-clamp recordings were acquired with Multiclamp 700B (Molecular Devices, San Jose, CA, USA) equipped with an A/D converter (BNC-2090, National Instruments, Austin, TX, USA or Digidata 1550B, Molecular Devices) and Igor Pro software version 4.01 (Wavemetrics, Portland, OR, USA) or pClamp 10 software (Molecular Devices) at 4 kHz. Series resistances were monitored, and the data were discarded when the series resistance changed by > 30 MΩ during recordings. mEPSCs were recorded at -70 mV in the presence of 1 μM tetrodotoxin and 100 μM picrotoxin, and mIPSCs were recorded at 0 mV in the presence of 1 μM tetrodotoxin, 10 μM

CNQX and 50 μM D-APV. mEPSCs and mIPSCs events above a threshold value (10 pA) were analyzed with Minianalysis software version 6.0.3 (Synaptosoft, Fort Lee, NJ, USA). For measurement of paired-pulse EPSCs, a bipolar stimulating electrode (FHC, Bowdoin, ME, USA) was placed in the stratum radiatum to stimulate Schaffer collaterals pathway. Pairs of evoked-EPSCs were recorded at a holding potential of -70 mV in the presence of 100 μM picrotoxin in the bath solution, and 2 mM QX-314 was added in internal solution. The pulse intensity was adjusted to 30–50% of the maximum amplitude. The stimulus frequency was 0.1 Hz. Inter-stimulus intervals of paired-pulse stimuli were set at 50 ms, 250 ms, 500 ms and 1000 ms.

qPCR

Total RNA was purified using the Qiagen RNeasy Plus Universal mini kit (QIAGEN, Hilden, Germany). Purified total RNA (0.5 μg) was subsequently reverse transcribed to cDNA using the ReverTra Ace qPCR RT kit (Toyobo, Osaka, Japan) according to the manufacturer's instructions. Real-time qPCR was performed with PowerUp SYBR Green Master Mix (Thermo Fisher Scientific, Waltham, MA, USA) in an applied Biosystems 7300 Real Time PCR System and relative expression was calculated via the 2Δ method and results were normalized to the internal control β-actin. The primers (sense and antisense, respectively) were as follows: mouse *Auts2*, 5'-AGAGCCTCTCACAGCCACTG-3' and 5'- GGTGGTGGGAGATGTGAGGA -3'; β-actin, 5'-GGCTGTATTCCCCTCCATCG-3', and 5'-CCAGTTGGTAACAATGCCATGT-3'.

RNA-sequencing and data analysis

Total RNA was extracted from hippocampal tissues of 4 control (*Auts2*$^{flox/flox}$) and 4 KO (*Emx1*$^{Cre/+}$;*Auts2*$^{flox/flox}$) mice at P14 using RNeasy Plus Universal Kit (QIAGEN, Hilden, Germany). Quality analyses and quantification of extracted RNA were performed using NanoDrop and Qubit Fluorometer (Thermo Fisher Scientific, Waltham, MA, USA), respectively. Sequencing libraries were prepared using the NEBNext Ultra Directional RNA Library Prep Kit for directional libraries (New England BioLabs, Tokyo, Japan) and the KAPA HTP Library Preparation Kits (KAPA Biosystems, Wilmington, MA, USA) according to the manufacturer's instructions. The RNA-seq libraries were sequenced (101 cycles) using the Illumina HiSeq platforms.

Raw sequence reads were aligned to the reference mouse genomes (GRCm38/mm10) by HISAT2 (Kim et al., 2015). Genome-wide expression levels were measured as a unit of transcripts

per kilobase million (TPM) using StringTie(Pertea et al., 2015) and the numbers of reads were counted per gene per sample using htseq-count within HTSeq (Anders et al., 2015). Finally, differentially expressed genes (DEGs) were identified by DESeq2 (Love et al., 2014). For gene ontology analysis, DAVID bioinformatics Resources 6.8 was used (National Institute of Allergy and Infectious Diseases-National Institute of Health; https://david.ncifcrf.gov). RNA-seq data has been deposited into GEO database with the accession number GSE134712.

Sample size and statistical analysis

Sample size was determined based on studies using established methods and on our previous experiments (Hori et al., 2015; Hori et al., 2014; Hori et al., 2005; Takahashi et al., 2012). Data analyses were performed blinded to the genotype. The number of samples and animals is indicated in the figure legends. All statistical analyses except transcriptome data processing and analysis were performed using GraphPad Prism 7 (GraphPad Software, La Jolla CA, USA). The normal distribution of data was confirmed by the Shapiro-Wilks test and if significant, a nonparametric Mann Whitney U test was used for comparison. Equal variance was tested by the F-test and when there was a significant difference, we used a two-tailed unpaired t-test with Welch's correction. When the data were within the assumptions of normal distribution and equal variance, a two-tailed unpaired t-test was used for comparison of the means between two groups. For comparison of more than 2 groups, a one-way analysis of variance (ANOVA) followed by the Dunnett's multiple comparison test was used.

In the behavioral analysis, two-way ANOVA followed by the Bonferroni test was used for multiple-group comparisons (reciprocal social interaction test and three-chamber social interaction test). Two-way ANOVA with repeated measurements followed by the Bonferroni test was used for multiple-group comparisons (locomotor activity, novel object recognition test, thigmotaxis and prepulse inhibition test).

Supplemental References

Anders, S., Pyl, P.T., and Huber, W. (2015). HTSeq--a Python framework to work with high-throughput sequencing data. Bioinformatics *31*, 166-169.

Erdmann, G., Schutz, G., and Berger, S. (2007). Inducible gene inactivation in neurons of the adult mouse forebrain. BMC Neurosci *8*, 63.

Harper, K.M., Hiramoto, T., Tanigaki, K., Kang, G., Suzuki, G., Trimble, W., and Hiroi, N. (2012). Alterations of social interaction through genetic and environmental manipulation of the 22q11.2 gene Sept5 in the mouse brain. Hum Mol Genet *21*, 3489-3499.

Harris, K.M., and Kater, S.B. (1994). Dendritic spines: cellular specializations imparting both stability and flexibility to synaptic function. Annu Rev Neurosci *17*, 341-371.

Hiramoto, T., Kang, G., Suzuki, G., Satoh, Y., Kucherlapati, R., Watanabe, Y., and Hiroi, N. (2011). Tbx1: identification of a 22q11.2 gene as a risk factor for autism spectrum disorder in a mouse model. Hum Mol Genet *20*, 4775-4785.

Hori, K., Nagai, T., Shan, W., Sakamoto, A., Abe, M., Yamazaki, M., Sakimura, K., Yamada, K., and Hoshino, M. (2015). Heterozygous Disruption of Autism susceptibility candidate 2 Causes Impaired Emotional Control and Cognitive Memory. PLoS One *10*, e0145979.

Hori, K., Nagai, T., Shan, W., Sakamoto, A., Taya, S., Hashimoto, R., Hayashi, T., Abe, M., Yamazaki, M., Nakao, K., *et al.* (2014). Cytoskeletal regulation by AUTS2 in neuronal migration and neuritogenesis. Cell reports *9*, 2166-2179.

Hori, K., Yasuda, H., Konno, D., Maruoka, H., Tsumoto, T., and Sobue, K. (2005). NMDA receptor-dependent synaptic translocation of insulin receptor substrate p53 via protein kinase C signaling. J Neurosci *25*, 2670-2681.

Ibi, D., Nagai, T., Koike, H., Kitahara, Y., Mizoguchi, H., Niwa, M., Jaaro-Peled, H., Nitta, A., Yoneda, Y., Nabeshima, T., *et al.* (2010). Combined effect of neonatal immune activation and mutant DISC1 on phenotypic changes in adulthood. Behav Brain Res *206*, 32-37.

Iwasato, T., Datwani, A., Wolf, A.M., Nishiyama, H., Taguchi, Y., Tonegawa, S., Knopfel, T., Erzurumlu, R.S., and Itohara, S. (2000). Cortex-restricted disruption of NMDAR1 impairs neuronal patterns in the barrel cortex. Nature *406*, 726-731.

Kikusui, T., Nakanishi, K., Nakagawa, R., Nagasawa, M., Mogi, K., and Okanoya, K. (2011). Cross fostering experiments suggest that mice songs are innate. PLoS One *6*, e17721.

Kim, D., Langmead, B., and Salzberg, S.L. (2015). HISAT: a fast spliced aligner with low memory requirements. Nature methods *12*, 357-360.

Koike, H., Ibi, D., Mizoguchi, H., Nagai, T., Nitta, A., Takuma, K., Nabeshima, T., Yoneda, Y., and Yamada, K. (2009). Behavioral abnormality and pharmacologic response in social isolation-reared mice. Behav Brain Res *202*, 114-121.

Lee, P.R., Brady, D.L., Shapiro, R.A., Dorsa, D.M., and Koenig, J.I. (2005). Social interaction

deficits caused by chronic phencyclidine administration are reversed by oxytocin. Neuropsychopharmacology *30*, 1883-1894.

Love, M.I., Huber, W., and Anders, S. (2014). Moderated estimation of fold change and dispersion for RNA-seq data with DESeq2. Genome Biol *15*, 550.

Metz, G.A., and Schwab, M.E. (2004). Behavioral characterization in a comprehensive mouse test battery reveals motor and sensory impairments in growth-associated protein-43 null mutant mice. Neuroscience *129*, 563-574.

Miyakawa, T., Yared, E., Pak, J.H., Huang, F.L., Huang, K.P., and Crawley, J.N. (2001). Neurogranin null mutant mice display performance deficits on spatial learning tasks with anxiety related components. Hippocampus *11*, 763-775.

Nadler, J.J., Moy, S.S., Dold, G., Trang, D., Simmons, N., Perez, A., Young, N.B., Barbaro, R.P., Piven, J., Magnuson, T.R., *et al.* (2004). Automated apparatus for quantitation of social approach behaviors in mice. Genes, brain, and behavior *3*, 303-314.

Nagai, T., Kitahara, Y., Shiraki, A., Hikita, T., Taya, S., Kaibuchi, K., and Yamada, K. (2010). Dysfunction of dopamine release in the prefrontal cortex of dysbindin deficient sandy mice: an in vivo microdialysis study. Neurosci Lett *470*, 134-138.

Nagai, T., Takuma, K., Kamei, H., Ito, Y., Nakamichi, N., Ibi, D., Nakanishi, Y., Murai, M., Mizoguchi, H., Nabeshima, T., *et al.* (2007). Dopamine D1 receptors regulate protein synthesis-dependent long-term recognition memory via extracellular signal-regulated kinase 1/2 in the prefrontal cortex. Learn Mem *14*, 117-125.

Pertea, M., Pertea, G.M., Antonescu, C.M., Chang, T.C., Mendell, J.T., and Salzberg, S.L. (2015). StringTie enables improved reconstruction of a transcriptome from RNA-seq reads. Nat Biotechnol *33*, 290-295.

Schindelin, J., Arganda-Carreras, I., Frise, E., Kaynig, V., Longair, M., Pietzsch, T., Preibisch, S., Rueden, C., Saalfeld, S., Schmid, B., *et al.* (2012). Fiji: an open-source platform for biological-image analysis. Nature methods *9*, 676-682.

Tachibana, R.O., Kanno, K., Okabe, S., Kobayasi, K.I., and Okanoya, K. (2020). USVSEG: A robust method for segmentation of ultrasonic vocalizations in rodents. PLoS One *15*, e0228907.

Tachibana, R.O., Oosugi, N., and Okanoya, K. (2014). Semi-automatic classification of birdsong elements using a linear support vector machine. PLoS One *9*, e92584.

Takahashi, H., Katayama, K., Sohya, K., Miyamoto, H., Prasad, T., Matsumoto, Y., Ota, M., Yasuda, H., Tsumoto, T., Aruga, J., *et al.* (2012). Selective control of inhibitory synapse development by Slitrk3-PTPdelta trans-synaptic interaction. Nat Neurosci *15*, 389-398, S381-382.

Takahashi, K., Nagai, T., Kamei, H., Maeda, K., Matsuya, T., Arai, S., Mizoguchi, H., Yoneda, Y., Nabeshima, T., Takuma, K., *et al.* (2007). Neural circuits containing pallidotegmental GABAergic neurons are involved in the prepulse inhibition of the startle reflex in mice. Biol Psychiatry *62*,

148-157.
Yang, M., and Crawley, J.N. (2009). Simple behavioral assessment of mouse olfaction. Curr Protoc Neurosci *Chapter 8*, Unit 8 24.
Yasumura, M., Yoshida, T., Yamazaki, M., Abe, M., Natsume, R., Kanno, K., Uemura, T., Takao, K., Sakimura, K., Kikusui, T., *et al.* (2014). IL1RAPL1 knockout mice show spine density decrease, learning deficiency, hyperactivity and reduced anxiety-like behaviours. Sci Rep *4*, 6613.
